# Supplementary material for: Doppler Indices of the Uterine, Umbilical and Fetal Middle Cerebral Artery in Diabetic versus Non-Diabetic Pregnancy: Systematic Review and Meta-Analysis
Source: Medicina (Kaunas). 2023 Aug 21;59(8):1502. doi: 10.3390/medicina59081502 (PMC10456372; doi:10.3390/medicina59081502)
Supplement: Supplementary file 1 [file medicina-59-01502-s001.zip › medicina-2491437-supplementary.pdf]

Supplementary material

Table S1. Search strategy

Database	Search strategy
PubMed	1st arm<br>(Diabetes, Gestational or Diabetes Mellitus, Gestational or Diabetes, Pregnancy-Induced or Gestational Diabetes) and (Color Doppler ultrasonography or Doppler or Doppler sonography or Doppler velocimetry or Pulse wave Doppler or pulsatility index or peak systolic velocity or systolic/diastolic ratio or S/D ratio or resistance index or resistive index or resistivity index)<br>2nd arm<br>(Diabetes Mellitus or Diabetes Mellitus, Type 1 or Diabetes Mellitus, Insulin-Dependent, 1 or Diabetes Mellitus, Type I or Type 1 Diabetes or Type 1 Diabetes Mellitus or Diabetes Mellitus, Type 2 or Diabetes Mellitus, Adult-Onset or Diabetes Mellitus, Type II or Type 2 Diabetes or Type 2 Diabetes Mellitus) and (pregnan*) and (Color Doppler ultrasonography or Doppler or Doppler sonography or Doppler velocimetry or Pulse wave Doppler or pulsatility index or peak systolic velocity or systolic/diastolic ratio or S/D ratio or resistance index or resistive index or resistivity index)
SCOPUS	1st arm<br>(TITLE-ABS-KEY(Diabetes, Gestational) OR TITLE-ABS-KEY(Diabetes Mellitus, Gestational) OR TITLE-ABS-KEY(Diabetes, Pregnancy-Induced) OR TITLE-ABS-KEY(Gestational Diabetes)) AND

	(TITLE-ABS-KEY(Color Doppler ultrasonography) OR TITLE-ABS-KEY(Doppler) OR TITLE-ABS-KEY(Doppler sonography) OR TITLE-ABS-KEY(Doppler velocimetry) OR TITLE-ABS-KEY(Pulse wave Doppler) OR TITLE-ABS-KEY(Pulsatility index) OR TITLE-ABS-KEY(Peak systolic velocity) OR TITLE-ABS-KEY(Systolic/diastolic ratio) OR TITLE-ABS-KEY(S/D ratio) OR TITLE-ABS-KEY(Resistance index) OR TITLE-ABS-KEY(Resistivity index))<br>2nd arm<br>(TITLE-ABS-KEY(Diabetes Mellitus) OR TITLE-ABS-KEY(Diabetes Mellitus, Type 1) OR TITLE-ABS-KEY(Diabetes Mellitus, Insulin-Dependent, 1) OR TITLE-ABS-KEY(Diabetes Mellitus, Type I) OR TITLE-ABS-KEY(Type 1 Diabetes) OR TITLE-ABS-KEY(Type 1 Diabetes Mellitus) OR TITLE-ABS-KEY(Diabetes Mellitus, Type 2) OR TITLE-ABS-KEY(Diabetes Mellitus, Adult-Onset) OR TITLE-ABS-KEY(Diabetes Mellitus, Type II) OR TITLE-ABS-KEY(Type 2 Diabetes) OR TITLE-ABS-KEY(Type 2 Diabetes Mellitus)) AND (TITLE-ABS-KEY(pregnan*)) AND (TITLE-ABS-KEY(Color Doppler ultrasonography) OR TITLE-ABS-KEY(Doppler) OR TITLE-ABS-KEY(Doppler sonography) OR TITLE-ABS-KEY(Doppler velocimetry) OR TITLE-ABS-KEY(Pulse wave Doppler) OR TITLE-ABS-KEY(Pulsatility index) OR TITLE-ABS-KEY(Peak systolic velocity) OR TITLE-ABS-KEY(Systolic/diastolic ratio) OR TITLE-ABS-KEY(S/D ratio) OR TITLE-ABS-KEY(Resistance index) OR TITLE-ABS-KEY(Resistivity index))

WOS	1st arm<br>(TS=(Diabetes, Gestational) OR TS=(Diabetes Mellitus, Gestational) OR TS=(Diabetes, Pregnancy-Induced) OR TS=(Gestational Diabetes)) AND (TS=(Color Doppler ultrasonography) OR TS=(Doppler) OR TS=(Doppler sonography) OR TS=(Doppler velocimetry) OR TS=(Pulse wave Doppler) OR TS=(Pulsatility index) OR TS=(Peak systolic velocity) OR TS=(Systolic/diastolic ratio) OR TS=(S/D ratio) OR TS=(Resistance index) OR TS=(Resistivity index))<br>2nd arm<br>(TS=(Diabetes Mellitus) OR TS=(Diabetes Mellitus, Type 1) OR TS=(Diabetes Mellitus, Insulin-Dependent, 1) OR TS=(Diabetes Mellitus, Type I) OR TS=(Type 1 Diabetes) OR TS=(Type 1 Diabetes Mellitus) OR TS=(Diabetes Mellitus, Type 2) OR TS=(Diabetes Mellitus, Adult-Onset) OR TS=(Diabetes Mellitus, Type II) OR TS=(Type 2 Diabetes) OR TS=(Type 2 Diabetes Mellitus)) AND (TS=(pregnan*)) AND (TS=(Color Doppler ultrasonography) OR TS=(Doppler) OR TS=(Doppler sonography) OR TS=(Doppler velocimetry) OR TS=(Pulse wave Doppler) OR TS=(Pulsatility index) OR TS=(Peak systolic velocity) OR TS=(Systolic/diastolic ratio) OR TS=(S/D ratio) OR TS=(Resistance index) OR TS=(Resistivity index))

Table S2. Quality assessment

Study	Selection domain	Comparability domain	Outcome/Exposure domain	Quality
Olofsson (1987) [29]	☆☆		☆☆	Poor
Landon (1989) [30]	☆☆		☆☆	Poor
Friedman (1989) [31]	☆☆		☆☆☆	Fair
Brown (1990) [32]	☆☆☆☆		☆☆☆	Good
Johnstone (1992) [33]	☆☆☆☆		☆☆☆	Good
Zimmermann (1992) [34]	☆☆☆		☆☆☆	Fair
Pachi (1993) [35]	☆		☆☆	Poor
Gagnon (1994) [36]	☆		☆☆	Poor
Weber (1994) [37]	☆☆☆		☆☆	Fair
Santolaya (1994) [38]	☆☆☆		☆☆	Fair
Gazzolo (1995) [39]	☆		☆☆	Poor
Saldeen (1996) [40]	☆		☆☆	Poor
Grunewald (1996) [41]	☆		☆☆	Poor
Weiner (1996) [42]	☆		☆☆	Poor
Ursem (1999) [43]	☆☆☆	☆	☆☆	Fair
Boito (2003) [44]	☆☆☆☆	☆☆	☆☆	Good
Tan (2005) [45]	☆☆☆☆	☆☆	☆☆	Good
Florio (2006) [46]	☆☆☆		☆☆	Fair
Girsen (2008) [47]	☆☆☆		☆☆	Fair
Russell (2009) [48]	☆☆☆		☆☆☆	Fair
To (2009) [49]	☆☆☆☆		☆☆☆	Good
Parlakgumus (2010) [50]	☆☆☆☆		☆☆☆	Good
Turan (2011) [51]	☆☆☆☆	☆	☆☆☆	Good
Nanda (2012) [52]	☆☆☆☆	☆	☆☆☆	Good
Fouda (2013) [53]	☆☆☆☆		☆☆☆	Good
Suranyi (2013) [54]	☆☆☆☆		☆☆	Fair
Savvidou (2013) [55]	☆☆☆☆		☆☆☆	Good
Shabani Zanjani (2013) [21]	☆☆☆☆	☆	☆☆	Good
Li (2014) [56]	☆☆☆☆		☆☆☆	Good
Gonzales Gonzales (2014) [57]	☆☆	☆	☆☆	Fair
Moran (2014) [58]	☆☆☆		☆☆☆	Fair

Bhorat (2014) [59]	☆☆☆☆	☆☆	☆☆☆	Good
Pala (2015) [60]	☆☆☆☆	☆☆	☆☆	Good
Liu (2016) [61]	☆☆☆☆		☆☆	Fair
Peixoto (2016) [14]	☆☆☆☆		☆☆☆	Good
Farshchian (2017) [62]	☆☆☆	☆☆	☆☆	Good
Bugatto (2017) [63]	☆☆		☆☆☆	Fair
Sweeting (2017) [64]	☆☆☆☆	☆	☆☆	Good
Meiramova (2018) [65]	☆☆☆☆		☆☆	Fair
Moodley (2018) [66]	☆☆☆		☆☆☆	Fair
Wong (2018) [67]	☆☆☆☆		☆☆	Fair
Ciobanu (2019) [22]	☆☆		☆☆	Poor
Dantas (2019) [68]	☆☆☆☆		☆☆	Fair
Bhorat (2019) [69]	☆☆☆☆	☆☆	☆☆	Good
Gasiorowska (2020) [70]	☆☆☆☆		☆☆	Fair
McLaren (2020) [71]	☆☆☆☆		☆☆	Fair
Bachani (2020) [72]	☆☆☆		☆☆	Fair
Tenenbaum-Gavish (2020) [73]	☆☆☆		☆☆☆	Fair
Lehtoranta (2020) [74]	☆☆		☆☆	Poor
Phadungkiatwattana (2021) [75]	☆☆		☆☆	Poor
Wei (2021) [18]	☆☆☆		☆☆	Fair
Zhang (2021) [76]	☆☆		☆☆	Poor
Alanyali (2021) [77]	☆☆		☆☆☆	Fair
Mecacci (2021) [78]	☆☆☆☆	☆☆	☆☆	Good
Liu (2021) [79]	☆☆☆		☆☆	Fair
Fatihoglu (2021) [80]	☆☆☆	☆	☆☆	Fair
Chen (2021) [81]	☆☆☆☆		☆☆	Fair
Ali Hssan (2021) [82]	☆☆		☆☆☆	Fair
Jamal (2021) [83]	☆☆☆☆		☆☆☆	Good
Perez-Martin (2022) [84]	☆☆☆☆		☆☆	Fair
Chatzakis (2022) [85]	☆☆☆		☆☆	Fair
Karaca Kutulmus (2022) [86]	☆☆☆☆	☆	☆☆	Good

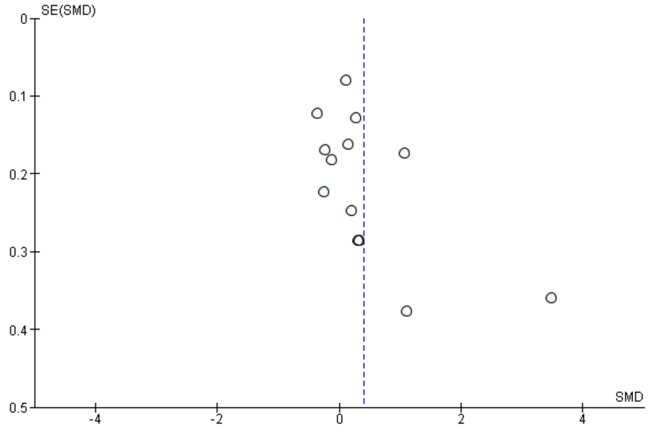

Figure S1. Funnel plot for UA-RI Doppler index in pregnant women with DM vs. pregnant women without DM.

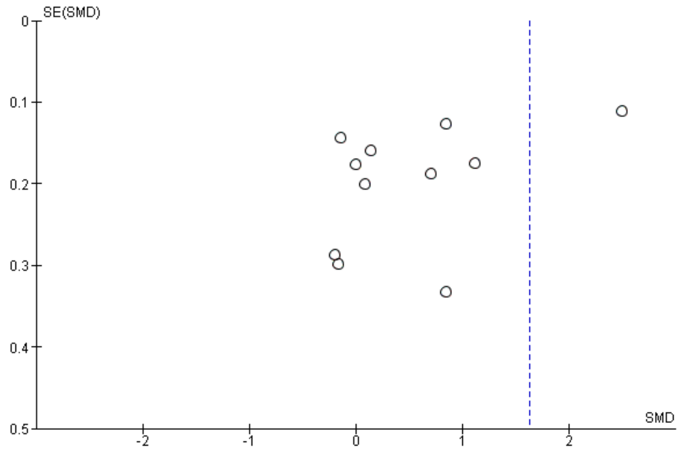

Figure S2. Funnel plot for UtA-PI Doppler index in pregnant women with DM vs. pregnant women without DM.

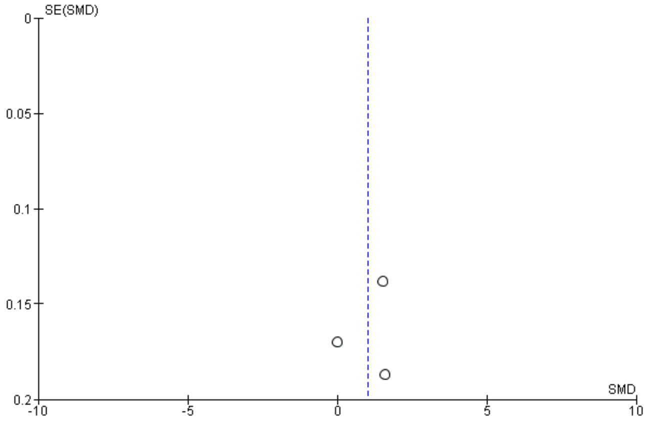

Figure S3. Funnel plot for UtA-S/D ratio Doppler index in pregnant women with DM vs. pregnant women without DM.

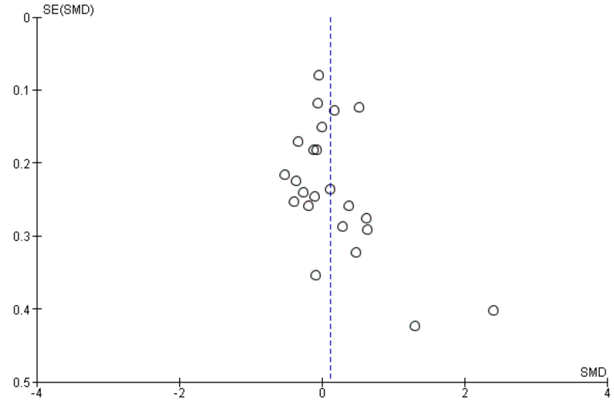

Figure S4. Funnel plot for UA-PI Doppler index in pregnant women with DM vs. women without DM.

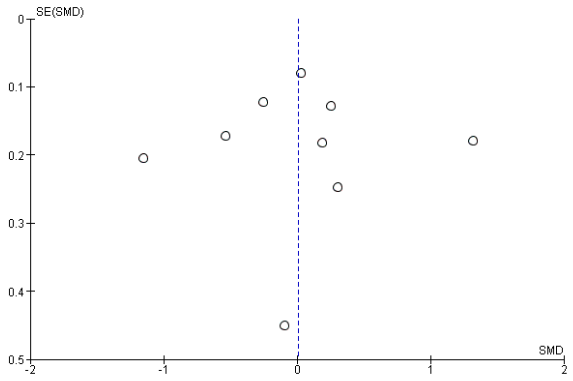

Figure S5. Funnel plot for UA-S/D ratio Doppler index in pregnant women with DM vs. women without DM.

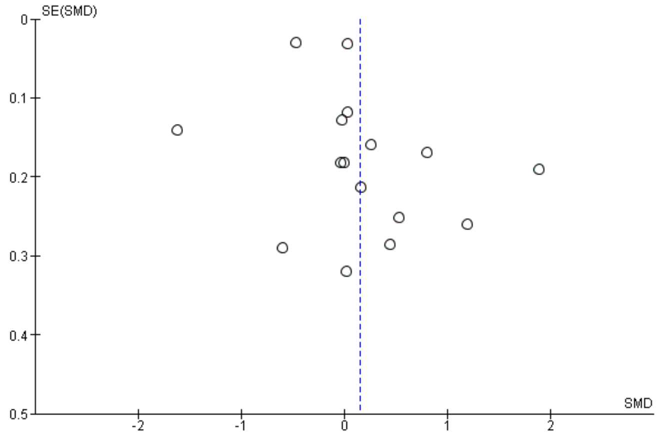

Figure S6. Funnel plot for MCA-PI Doppler index in pregnant women with DM vs. women without DM.

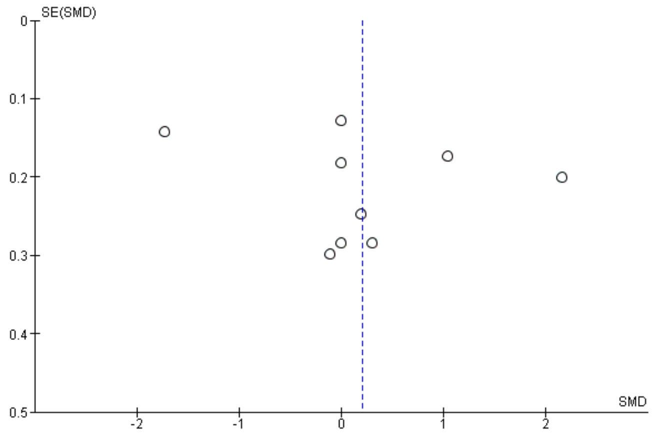

Figure S7. Funnel plot for MCA-RI Doppler index in pregnant women with DM vs. women without DM.

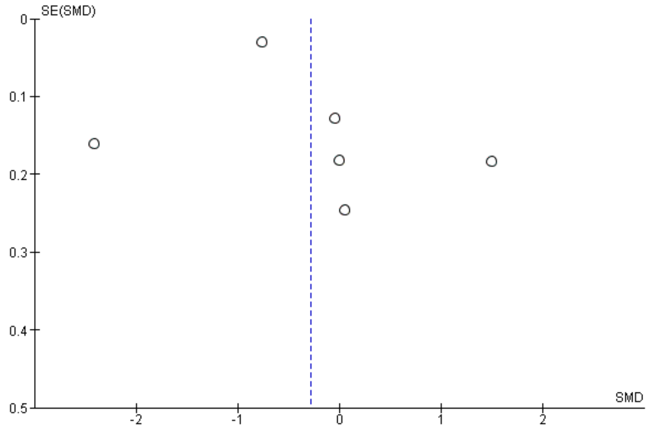

Figure S8. Funnel plot for MCA-S/D ratio Doppler index in pregnant women with DM vs. women without DM.

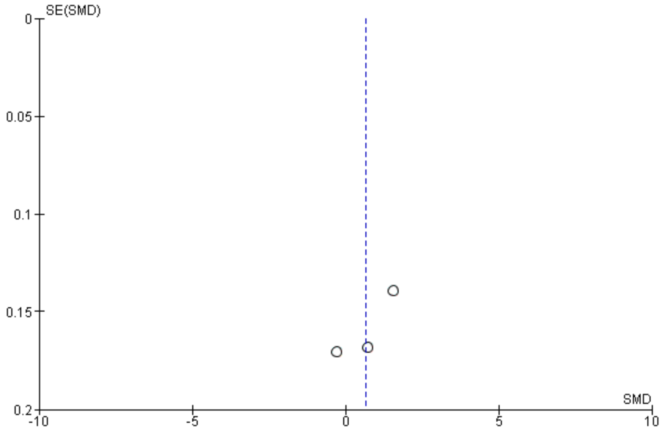

Figure S9. Funnel plot for UtA-RI Doppler index in pregnant women with DM vs. women without DM.

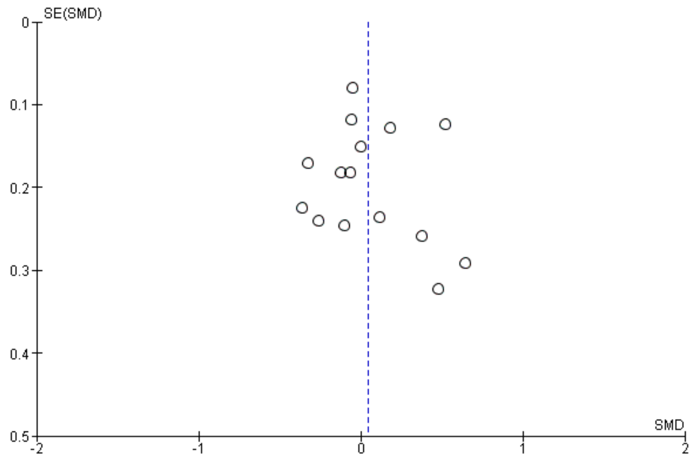

Figure S10. Funnel plot for UA-PI Doppler index in pregnant women with GDM vs. women without GDM.

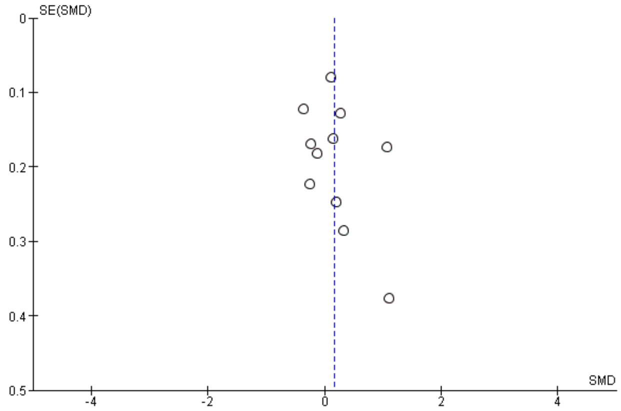

Figure S11. Funnel plot for UA-RI Doppler index in pregnant women with GDM vs. women without GDM.

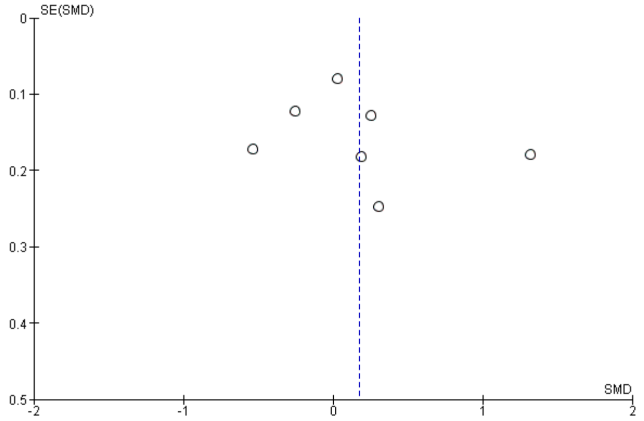

Figure S12. Funnel plot for UA-S/D ratio Doppler index in pregnant women with GDM vs. women without GDM.

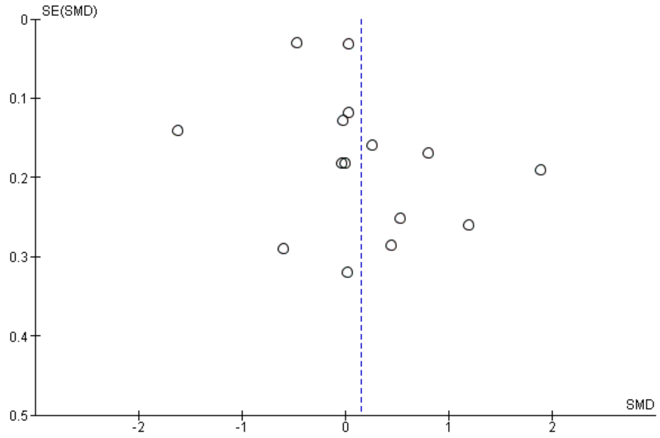

Figure S13. Funnel plot for MCA-PI Doppler index in pregnant women with GDM vs. women without GDM.

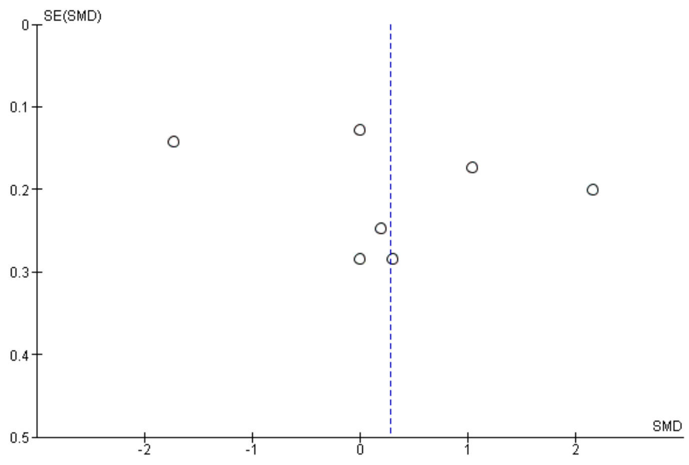

Figure S14. Funnel plot for MCA-RI Doppler index in pregnant women with GDM vs. women without GDM.

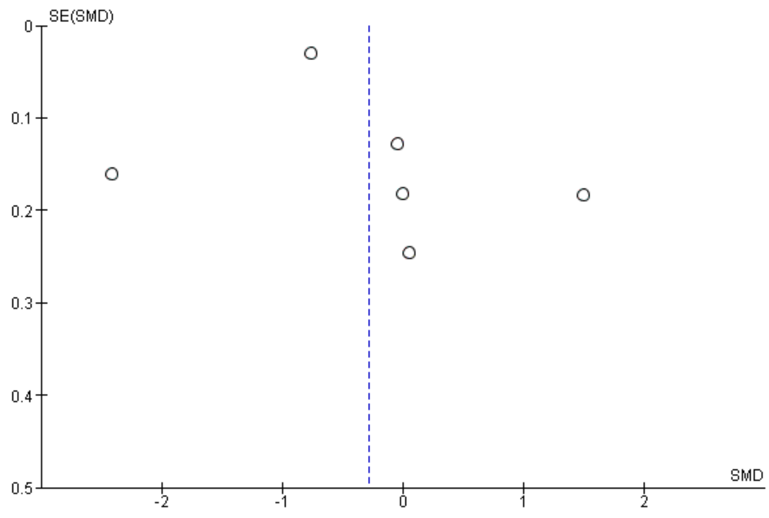

Figure S15. Funnel plot for MCA-S/D ratio Doppler index in pregnant women with GDM vs. women without GDM.

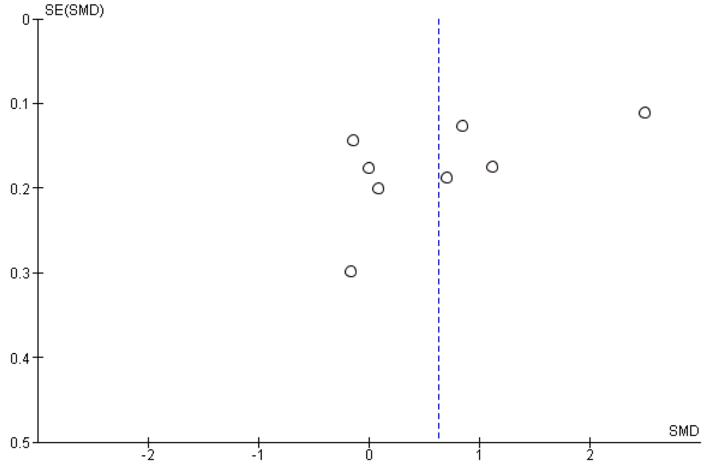

Figure S16. Funnel plot for UtA-PI Doppler index in pregnant women with GDM vs. women without GDM.

Table S3. Definitions and criteria of pregestational and gestational diabetes mellitus used in included studies

Author, year	DM type (White classification)	Diabetes mellitus definition	Diabetes mellitus criteria
Olofsson (1987) [29]	DM	NR	NR
Landon (1989) [30]	DM	NR	**WHITE**
Friedman (1989) [31]	DM	NR	NR
Brown (1990) [32]	GDM	NR	NR
Johnstone (1992) [33]	DM type 1 (B, C, D, F/R) GDM (A2)	Conservative criteria were used for initiating insulin treatment in gestational diabetics, that is, patients had an abnormal 75 g glucose tolerance test (usually plasma glucose > 11.2mmol/l at 2 h) and after treatment with diet still had pre-prandial glucose levels persistently >6.5mmol/l.	**WHITE**
Zimmermann (1992) [34]	DM type 1 (B,C,D, F/R)	Mean blood glucose values of 6.3mmol/l. The upper limit of normal is set at 6.5% of total hemoglobin.	**WHITE**
Pachi (1993) [35]	DM type 1 (B, C, D, R)	A mean capillary glucose value > 6.1mmol/l (110 mg/dl) was selected to indicate poor glucose control.	**WHITE**
Gagnon (1994) [36]	DM type 1 DM type 2 GDM	GDM - 50-g oral glucose load in a fasted state and was considered negative if the plasma glucose value 1 hr after the glucose load was <7.2mmol/l (130 mg/dl).	**National Diabetes Data Group**
Weber (1994) [37]	DM type 1 (B, C, D, F, RF)	NR	**WHITE**
Santolaya (1994) [38]	GDM	One-hour 50g glucose tolerance test for screening at their initial visit and at 28 gestational weeks. If it was abnormal, a three-hour glucose tolerance test was performed.	NR
Gazzolo (1995) [39]	GDM	A 3-h oral glucose tolerance test (OGTT) was performed with a 100-g oral glucose load and plus 2 S.D. defined the upper limits of normality during each hourly testing period. The diagnosis was made when two or more venous plasma glucose concentrations met or exceeded the following glucose serum levels: fasting level of 105 mg/dl; 1-h of 190 mg/dl; 2-h of 165 mg/dl; and 3oh of 145 mg/dl. The test was performed between 24 and 28 weeks of gestation.	**O`Sullivan test as initial screening of the National Diabetes Data Group accepted by the Spanish Group of Diabetes in Pregnancy**
Saldeen (1996) [40]	DM type 2 GDM	A 2-h glucose concentration of > 11.1mmol/L is regarded as diabetes and a value of 7.8-11.0 as impaired glucose tolerance.	**WHO**

Grunewald (1996) [41]	DM type 1	NR	NR
Weiner (1996) [42]	DM type 1<br>(B, C, D, F, R)<br>GDM (A)	The goal of treatment was to maintain fasting maternal blood glucose levels below 100 mg% and two<br>hour postprandial levels below 120 mg%. Only well controlled diabetics with mean blood glucose levels below 95 rag% formed the study group. In all these patients maternal blood HbAlc was below 6.3%.	**WHITE**
Ursem (1999) [43]	DM type 1 (B, C, R, F/R)	NR	**WHITE**
Saldeen (1996) [40]	DM type 2<br>GDM	After a 75 g oral glucose tolerance test, a 2 h glucose concentration of >11.0mmol/L was regarded as diabetes and a value of 7.8–11.0 as IGT.	**WHO**
Boito (2003) [44]	DM type 1 (B, C, D, R, F/R)	NR	**WHITE**
Tan (2005) [45]	DM type 1<br>DM type2<br>GDM	Fasting venous plasma glucose level of> 7.0mmol/L and a 2-h post glucose load of<br>>11.1mmol/L after a 75 g modified glucose tolerance test; impaired glucose tolerance: fasting venous<br>plasma sugar level <7.0mmol/L and a 2-h post glucose load between 7.8mmol/L and 11.1mmol/L.	**WHO**
Florio (2006) [46]	GDM (A1)	NR	**WHITE**
Girsen (2008) [47]	DM type 1 (B, C, D, F)		**WHITE**
Russell (2009) [48]	DM type 1 (B, C, D, F, R, F/R)	NR	**WHITE**
To (2009) [49]	GDM	Patients with risk factors for GDM received a 75 g oral glucose tolerance test (OGTT) at 24 weeks gestation or before. Low-risk patients underwent a random glucose test between 24 to 28 weeks, and those with venous plasma glucose values 6.0mmol/ l would in turn received a full OGTT. All diagnoses of GDM were based on 75 g OGTT, and consecutive cases with GDM (fasting glucose levels 47.0 and 2-h glucose value 11.1mmol/l) and IGT (2-h glucose level 7.8 to 11mmol/l).	**WHO**
Parlakgumus (2010) [50]	DM type 1<br>DM type 2<br>GDM	50 g glucose tolerance test at 24gw	NR
Turan (2011) [51]	DM	NR	NR

Nanda (2012) [52]	GDM	Screening for GDM in our hospital is based on a 2-step approach. In all women, random plasma glucose is measured at 24 to 28 weeks of gestation; and if the concentration is more than 6.7mmol/L, a 100-g oral glucose tolerance test is carried out within the subsequent 2 weeks. The diagnosis of GDM is made if the fasting plasma glucose level is at least 6mmol/L or if the plasma glucose level 2 hours after the oral administration of 75 g glucose is 7.8mmol/L or more.	NR
Fouda (2013) [53]	DM type 1<br>DM type 2	Fasting plasma glucose level at or above 126mg/dL, plasma glucose at or above 200mg/dL two hours<br>after a 75 g oral glucose load as in a glucose tolerance test, symptoms of hyperglycemia and casual plasma glucose at or above 200mg/dL.	American diabetes association (**ADA**)
Suranyi (2013) [54]	DM type 1 (B, C, D)<br>GDM (A1, A2)	NR	**WHO**
Savvidou (2013) [55]	GDM	NR	**National Institute for Health and Clinical<br>Excellence (NICE guidelines)**
Li (2014) [56]	GDM	Plasma glucose concentrations were measured at 0, 60, and 120 minutes when the woman received at 75 g OGTT in the second trimester of pregnancy. GDM was diagnosed when patient plasma glucose levels exceeded or reached one of the following thresholds: fasting glucose ≥5.6mmol/L; one hour glucose ≥10.3mmol/L or two hour glucose ≥8.6mmol/L.	NR
Gonzales Gonzales (2014) [57]	DM type 1<br>DM type 2	NR	
Shabani Zanjani (2013) [21]	GDM	IGT was diagnosed if OGTT 2 h glucose value was 8.0–10.9mmol/L and diabetes mellitus was diagnosed if OGTT 2 h glucose value was 11.0mmol/L or more.	**WHO**
Moran (2014) [58]	DM type 1<br>DM type 2	NR	
Bhorat (2014) [59]	GDM (A2)	NR	NR
Pala (2015) [60]	GDM	75g OGTT was preferred for GDM diagnostic criteria. GDM diagnosis was made when<br>one or more of the values of three maternal blood samples were high (fasting ≥92, first hour ≥180, second hour ≥153 mg/dl).	The International Association of Diabetes in Pregnancy Study Groups (**IADPSG**).
Liu (2016) [61]	GDM	Plasma glucose concentrations were measured at 0, 60, and 120 min after the woman received a 75 g OGTT in the second trimester of pregnancy. GDM was diagnosed when the patient's plasma glucose levels exceeded or reached one of the following thresholds: fasting glucose level ≥5.1mmol/L; 1-h glucose level ≥10.0mmol/L; and 2-h glucose level ≥8.5mmol/L.	The American Diabetes Association (**ADA**).
Peixoto (2016) [14]	GDM	One or more of the following criteria: fasting glucose test >92mg/dL, 1-h 75-g glucose challenge test >180mg/dL and 2-h 75-g glucose challenge test >153mg/dL	The International Association of Diabetes and Pregnancy Study Groups (**IADPSG**).

Farshchian (2017) [62]	DM<br>GDM	NR	NR
Bugatto (2017) [63]	GDM (A1, A2)	Screening was performed using a 2-step approach in pregnant women between 24 and 28 weeks of gestation. The initial screening procedure consisted of a 50-g glucose challenge test, with a 1-hour blood glucose cutoff set at ≥ 140mg/dL (7.76mmol/l). Women with a positive screening test underwent a confirmatory 3-hour, 100-g oral glucose tolerance test (fasting glucose ≥105mg/dL [5.82mmol/L; 1-hour, ≥190mg/dL (10.54mmol/L); 2-hour, ≥165mg/dL [9.15mmol/L]; and 3-hour, ≥145mg/dL [8.04mmol/L]). Gestational diabetes mellitus was defined when 2 or more plasma glucose measurements were equal or higher than the cutoff points.	**O`Sullivan test as initial screening of the National Diabetes Data Group accepted by the Spanish Group of Diabetes in Pregnancy**<br><br>American Diabetes Association (**ADA**)
Sweeting (2017) [64]	GDM	GDM was evaluated between 24 to 28 weeks' gestation with the 75 gram 2-h OGTT; or the OGTT following a positive screening GCT. GDM was classified as either 'early GDM' or 'standard GDM' depending on nwhether diagnosed < or ≥24 weeks' gestation, respectively. Women at high-risk for GDM (ie. non-Caucasian ethnicity, age ≥40 years, body mass index (BMI) ≥25 kg/m2, family history of (type 2) diabetes, previous GDM or macrosomia, polycystic ovary syndrome (PCOS) or prescribed medication associated with hyperglycaemia), were advised to undergo early testing for GDM with an OGTT generally soon after the first antenatal appointment; repeated at 18 to 20 weeks' and 24 to 28 weeks' gestation if still negative. Diagnostic criteria did not change during the study period.	The Australian Diabetes in Pregnancy (**ADIPS**).
Meiramova (2018) [65]	GDM	Blood glucose level ≥5.1 mmol/L on fasting state.	NR
Moodley (2018) [66]	DM<br>GDM	NR	NR
Wong (2018) [67]	GDM	Screening for GDM was universally performed at 24 to 28 gestational weeks. Plasma glucose was measured 1 hour after a 50-g glucose challenge test (GCT) in a non-fasting state, and if the glucose level was more than 140 mg/dL the women then received a 3-hour 100-g oral glucose tolerance test (OGTT). GDM was diagnosed if two or more glucose levels exceeded the given threshold, including fasting plasma glucose level ≥ 105 mg/dL, plasma glucose level 1 hour after OGTT ≥ 190 mg/dL, plasma glucose level 2 hours after OGTT ≥ 165 mg/dL, and plasma glucose level 3 hours after OGTT ≥ 145 mg/dL.	**The National Diabetes Data Group**
Ciobanu (2019) [22]	DM<br>DM type 1<br>DM type 2	NR	NR
Dantas (2019) [68]	GDM	Diagnosis was made using an OGTT. This test confirmed a diagnosis of GDM when the fasting 75-g OGTT was greater than 5.1 mmol/L; the 1-hr OGTT was greater than 9.9 mmol/L; and the 2-hr OGTT was greater than 8.4 mmol/L.	The American Diabetes Association (**ADA**)
Bhorat (2019) [69]	GDM	2-h level > 7.8–11 mmol/l after a 75 g OGTT	**WHO**

Gasiorowska (2020) [70]	DM	NR	NR
McLaren (2020) [71]	DM<br>GDM	Pregestational diabetes was defined as an HbA1C of 6.5%, a random blood glucose greater than 200 mg/dL, and/or fasting glucose of ≥126 mg/dL. Definition for gestational diabetes - NR.	NR
Bachani (2020) [72]	GDM	NR	NR
Tenenbaum-Gavish (2020) [73]	GDM	Diagnosis of GDM was made in the second trimester using the standard two-step protocol: At 24–28 weeks' gestation, a 1-h GCT (50 g) was performed and if the glucose level exceeded 140 mg/dL, a 3-h<br>OGTT (100 g) was carried out. GDM was diagnosed in women who had two or more abnormal OGTT results according to Carpenter and Costan: fasting ≥95 mg/dL (5.2 mM, 1-h ≥180 mg/dL (10 mM), 2-h<br>≥155 mg/dL (8.6 mM), 3-h ≥140 mg/dL (7.8 mM).	The American College of Obstetricians and Gynecologists (**ACOG**) **2013.**<br>Carpenter MW, Coustan DR. Criteria for screening tests for gestational diabetes. Am J Obstet Gynecol. 1982 Dec 1; 144 (7): 768-73.
Lehtoranta (2020) [74]	DM type 1	NR	NR
Phadungkiatwattana (2021) [75]	DM<br>GDM	GDM was diagnosed before 28 weeks of gestation with one or more of the glucose levels meeting the following thresholds after a 75-g OGTT: fasting glucose ≥ 92 mg/dL, 1 hour ≥ 180 mg/dL, 2 hour ≥153 mg/Dl.	**Fifth International Workshop-Conference on Gestational Diabetes**
Wei (2021) [18]	GDM	NR	NR
Zhang (2021) [76]	GDM	Screened with 50 g of glucose at the 25th–28th weeks. If the blood glucose level was higher than 7.9mmol/L after 1 hour, the patient was confirmed to be positive of glucose screening<br>and then examined for fasting blood glucose. If the blood glucose level was abnormal, the patient was diagnosed as having GDM. The healthy pregnant women underwent an OGTT. Further, 75 g<br>OGTT was performed, with 5.6 mmol/L being the fasting blood glucose content upper limit under normal conditions. The blood glucose content 1 hour, 2 hours, and 3 hours after a meal was 10.3 mmol/L, 8.6 mmol/L, and 6.7 mmol/L, respectively. If the content of two or more terms exceeded the normal range, the patient was diagnosed as GDM.	**Sixth edition of Obstetrics and Gynecology**<br>(People's Medical Publication House)
Alanyali (2021) [77]	DM<br>DM type 1<br>DM type 2	NR	NR
Mecacci (2021) [78]	DM type 1	NR	**WHO**
Liu (2021) [79]	GDM	All non-diabetic pregnant women routinely undergo 75g OGTT at<br>24 to 28 weeks of gestation, meeting or exceeding at least one of the following indicators:<br>fasting blood glucose, after taking sugar 1 The 2-hour blood glucose was 5.1 mmol/L,<br>10.0 mmol/L and 8.5 mmol/L, respectively, which was the diagnosis of GDM.	The International Association of the Diabetes and Pregnancy Study Groups **(IADPSG) 2010.**

Fatihoglu (2021) [80]	GDM	Oral glucose tolerance test (OGTT) and HbA1C values obtained at 24–28 weeks of gestation.	The American Diabetes Association (**ADA**).
Chen (2021) [81]	GDM	75-g oral glucose tolerance test (OGTT); GDM was diagnosed at 24–28 weeks when one or more of the venous plasma glucose measurements met or exceeded the following thresholds after a 75-g OGTT: fasting blood glucose ≥ 5.1 mmol/L (92 mg/dl); 1 h plasma glucose level ≥ 10.0 mmol/L (180 mg/dl); or 2 h plasma glucose level ≥ 8.5 mmol/L (153 mg/dl).	The International Association of the Diabetes and Pregnancy Study Groups (**IADPSG**).
Ali Hssan (2021) [82]	GDM	Two-hour oral glucose tolerance test (OGTT): glucose levels of 95, 140, and 120 mg/dl for fasting; one hour and two-hour respectively post 75 g glucose.	The American Diabetes Association (**ADA**) 2016.
Jamal (2021) [83]	GDM	Two-hour oral glucose tolerance test at 24-28 wk of gestational age. A diagnosis was made when any of the following was met or exceeded: fasting glucose level of 92 mg/dl, one-hr level of 180 mg/dl, and/or two-hr level of 153 mg/dl.	The American Diabetes Association (**ADA**) 2018.
Perez-Martin (2022) [84]	GDM	The O'Sullivan's test was used as an initial screening test (fasting blood glucose and 1 hour after administration of 50 g of glucose) and an oral glucose tolerance test (OGTT) as a confirmatory test (two or more pathological values in the determination of fasting blood glucose and 1, 2 and 3 h after administration of 100 g of glucose).	**O`Sullivan test as initial screening of the National Diabetes Data Group accepted by the Spanish Group of Diabetes in Pregnancy**
Chatzakis (2022) [85]	GDM	One blood glucose value equal or greater than either 92 mg/dL, or 180 mg/dL, or 153 mg/dL, at either fasting, or 60 min or 120 min, respectively, after consumption of 75 g of glucose secured positive diagnosis.	The International Association of Diabetes and Pregnancy Study Groups (**IADPSG**).
Karaca Kutulmus (2022) [86]	GDM	The diagnosis of gestational diabetes mellitus was made between 24 and 28 weeks of gestation based on a 75 g oral glucose tolerance test which was performed on both the study and the control groups for gestational diabetes mellitus screening.	**NICE guidelines**. Diabetes in pregnancy: management from preconception to the postnatal period, 2015.

Table S4. Inclusion and exclusion criteria used in included studies

Author, year	Multiple pregnancy	Nulliparity	HTA	PIH	PE	Cardiovascular diseases	Smoking	Renal diseases	Obesity	Other exclusion criteria	Other inclusion criteria
Olofsson (1987) [29]	NR	No	No	No	NR	NR	NR	NR	NR	NR	NR
Landon (1989) [30]	NR	NR	No	No	No	NR	NR	NR	NR	NR	NR
Friedman (1989) [31]	Yes	NR	NR	No	NR	NR	NR	NR	NR	Growth retardation, arrhythmias	NR
Brown (1990) [32]	NR	No	NR	No	NR	NR	NR	NR	NR	NR	NR
Johnstone (1992) [33]	NR	NR	No	No	No	No	NR	NR	NR	NR	Auto-immune thyroiditis 5, Epilepsy2 Sarcoidosis 2 Addison's disease 1 Recurrent pancreatitis 1 Myocardial infarction 1
Zimmermann (1992) [34]	NR	NR	No	No	No	NR	NR	No	No	NR	NR
Pachi (1993) [35]	NR	No	No	No	No	NR	NR	No	Yes	No ultrasonographic assessment of gestational age during the first trimester of pregnancy	Weekly ambulatory glucose profiles
Gagnon (1994) [36]	NR	NR	No	NR	No	No	No	No	No	If only one abnormal value was found	NR

Weber (1994) [37]	NR	NR	NR	No	No	NR	NR	NR	NR	NR	NR
Santolaya (1994) [38]	NR	No	Yes	NR	NR	NR	NR	NR	No	NR	NR
Gazzolo (1995) [39]	NR	NR	NR	NR	NR	NR	NR	NR	NR	NR	NR
Saldeen (1996) [40]	NR	NR	Yes	Yes	Yes	NR	No	NR	NR	NR	NR
Grunewald (1996) [41]	NR	No	No	No	No	NR	NR	No	No	NR	NR
Weiner (1996) [42]	NR	NR	NR	No	NR	NR	NR	NR	NR	NR	NR
Ursem (1999) [43]	Yes	No	NR	NR	NR	NR	NR	NR	NR	NR	NR
Boito (2003) [44]	Yes	No	NR	NR	NR	NR	No	NR	NR	NR	NR
Tan (2005) [45]	Yes	NR	Yes	NR	NR	NR	NR	Yes	NR	Pre-existing medical conditions, such as connective tissue disease, ultrasonographic evidence of gross fetal abnormalities and IUGR from other causes.	NR
Florio (2006) [46]	Yes	No	NR	Yes	Yes	NR	NR	NR	NR	Preterm labor or infection	NR
Girsen (2008) [47]	NR	NR	Yes	Yes	Yes	NR	NR	NR	NR	Placental insufficiency and fetal acidemia during delivery, as well as pregnancies with fetal structural and chromosomal abnormalities	normal umbilical artery (UA) blood velocity waveform pattern for gestational age and UA blood sample taken at delivery
Russell (2009) [48]	NR	No	No	No	No	No	No	No	No	NR	NR
To (2009) [49]	NR	No	No	No	No	NR	NR	NR	No	Patients booked after 24 weeks who were not exposed to the GDM screening program	NR

Parlakgumus (2010) [50]	Yes	NR	Yes	Yes	Yes	Yes	Yes	Yes	Yes	Chronic diseases other than diabetes, who were on medications other than insulin and vitamins, pregnancies with fetuses with known or suspected chromosomal and structural anomalies and intrauterine infection	NR
Turan (2011) [51]	NR	NR	NR	NR	NR	NR	NR	NR	No	Fetal cardiac anomalies diagnosed at the 1st trimester, a reversed a-wave in the ductus venosus and the presence of tricuspid regurgitation and extracardiac fetal anomalies.	a crown–rump length of 45–84 mm, a normal nuchal translucency thickness below the 95th centile, normal fetal anatomy and normal ductus venosus and tricuspid valve Doppler waveforms
Nanda (2012) [52]	NR	No	No	No	No	NR	No	NR	No	NR	NR
Fouda (2013) [53]	Yes	No	No	No	No	Yes	NR	Yes	Yes	Ultrasound examination done more than one week before delivery, diabetic vasculopathy, preexisting medical conditions, such as connective tissue disease, uncertainty of the date of the last menstrual period, major fetal abnormalities and absence of end diastolic waveform or reverse waveform	NR

Suranyi (2013) [54]	Yes	NR	NR	NR	NR	NR	Yes	NR	NR	Enlarged (>3mm) nuchal translucency from 11^{+0} to 13^{+6} gw, fetal or neonatal structural or chromosomal anomaly, inadequate localization of the placenta (placenta praevia and posterior placenta), self-reported drugs, alcohol, caffeine abuse, exposure to circulatory medication (oxerutins and calcium dobesilate), or not signing the consent form. Diabetes accompanied by another systemic disease (autoimmune disease, vasculitis, hemophilia, thrombophilia, HIV infection, etc.).	NR
Savvidou (2013) [55]	Yes	No	No	No	No	No	No	No	No	Missing outcome data, ending in miscarriage or termination, fetal defects, delivery before 30gw because they may not have had screening and diagnosis of GDM, and prepregnancy DM	Singleton pregnancy and delivery of a phenotypically normal neonate at or after 30gw
Li (2014) [56]	Yes	No	No	No	No	NR	NR	NR	No	NR	Newborn without congenital anomalies, singleton pregnancy, no HIV and no syphilis, and OGTT performed in the 2nd trimester.
Gonzales Gonzales (2014) [57]	No	No	No	No	No	Yes	No	Yes	No	pre-existing maternal diseases (other than DM or chronic hypertension), any alcohol or drug use and any severe or lethal fetal malformations, fetal aneuploidy	

Shabani Zanjani (2013) [21]	Yes	No	Yes	Yes	Yes	NR	NR	NR	NR	History of diabetes mellitus, blood disorders and hyperlipidemia any other well-known condition affecting fetal blood flow velocity such as fetal abnormalities of any kind (intrauterine growth restriction (IUGR), macrosomia, etc.), and obstetric history of previous complications (IUGR and placental abruption)	pregnant woman with at least 24 weeks of gestation
Moran (2014) [58]	NR	No	Yes	Yes	Yes	NR	NR	NR	No	Medical disorder requiring treatment, fetal anomaly or a suspicion or diagnosis of IUGR	NR
Bhorat (2014) [59]	No	NR	NR	NR	Yes	NR	NR	NR	NR	Exclusion criteria were multiple pregnancies, congenital malformations, evidence of placental-mediated disease and abnormal fetal heart rates (either tachycardia or bradycardia). Placental-mediated disease was defined by either the presence of growth restriction (AC <10th percentile for gestational age with an elevated UA resistance index (RI) >90th percentile for gestational age) and/or presence of preeclampsia.	NR
Pala (2015) [60]	Yes	No	Yes	NR	NR	Yes	Yes	Yes	NR	Type I and Type II DM, polycystic ovary	NR

										syndrome, hyperlipidemia before pregnancy, metabolic syndrome history, drugs use (which might affect blood glucose and insulin levels), endocrine disorders, liver, and chronic systemic inflammatory and infectious diseases, and malignancies, as well as pregnancies whose placenta could not be seen entirely and with the evidence of fetal chromosomal or structural anomaly	
Liu (2016) [61]	NR	NR	NR	Yes	Yes	NR	Yes	Yes	NR	Well-known condition affecting fetal blood flow, such as IUGR, anemia, and hypoxemia, history of a newborn with congenital anomalies, history of diabetes mellitus, blood disorders, or hyperlipidemia, HIV and syphilis, and history of drinking	NR
Peixoto (2016) [14]	Yes	No	Yes	NR	NR	NR	NR	Yes (chronic renal diseases)	NR	Pregestational DM and collagenoses as systemic erythematosus lupus	Singleton pregnancies, gestational age determined by the last menstrual period and confirmed by first-trimester ultrasound using crown-rump length until 13th week, absent of fetal structural malformations or chromosomal abnormalities in the ultrasound exam and

											confirmed in the postnatal period
Farshchian (2017) [62]	Yes	NR	Yes	NR	NR	NR	NR	NR	NR	NR	No intrauterine growth retardation, singleton pregnancy, and not taking medications such as insulin
Sweeting (2017) [64]	Yes	No	No	No	No	NR	No	NR	NR	Ending pregnancy before the traditional screening point for GDM (24 to 28gw), including miscarriage, termination of pregnancy for fetal chromosomal or structural abnormality or pre-term delivery. Pregestational diabetes, missing clinical/outcome data and where GDM was diagnosed based on a 50 g 1-h GCT alone	Singleton pregnancy, received antenatal care and delivered in the same maternity unit
Meiramova (2018) [65]	NR	NR	Yes	NR	NR	NR	NR	NR	No	Maternal age <18years, pregnancy after in vitro fertilization, pre-pregnancy disorders of carbohydrate metabolism	Pregnant women 18-42 years of Kazakh nationality between 30-32gw

Moodley (2018) [66]	Yes	NR	No	Yes	Yes	Yes (maternal history of congenital heart disease, given the reported potentially abnormal uteroplacental blood flow in these patients)	NR	NR	No	1) in vitro fertilization pregnancy, 2) conditions that could affect arterial stiffness such as maternal antibody mediated rheumatologic disease including systemic lupus erythematosus and Sjogren's syndrome, 3) known fetal extra-cardiac or chromosomal abnormalities, 4) fetal hydrops, 5) intrauterine growth restriction (IUGR), 6) maternal food ntake within 3 hours prior to testing due to effects on augmentation index, and 7) gestational hypertension	1)pregnant women between the ages of 18-40 years, 2) 20-28 weeks gestation, 3) single gestation pregnancy, 4) normal fetal cardiac structures, including normal thickness of the interventricular septum, on the fetal echocardiogram performed at the initial visit and 5) referral for gestational or pre-gestational diabetic pregnancy
Wong (2018) [67]	Yes	No	Yes	Yes	Yes	NR	NR	NR	Yes (BMI≥25 kg/m2)	fetal chromosomal or structural anomalies detected during karyotyping or sonographic examinations, pregestational DM, and multifetal pregnancy	maternal age ≥ 35 years, previously given birth to a macrosomic infant (birth weight ≥ 4000 g), previous history of GDM, and family history of DM in first- or second-degree relatives
Dantas (2019) [68]	Yes	NR	Yes	NR	NR	NR	Yes	NR	NR	maternal fetal pathology that might affect ultrasonographic parameters (e.g. arterial hypertension) was excluded from the present study	age older than 18 years, no fetal malformation
Bhorat (2019) [69]	Yes	NR	NR	NR	Yes	NR	NR	NR	NR	pregestational diabetes mellitus, congenital malformations, evidence of placental-mediated disease (defined by either the presence of growth restriction (AC < 10th percentile for	NR

										gestational age with an elevated umbilical artery resistance index > 90th percentile for gestational age) and/or the presence of preeclampsia as defined by a blood pressure of greater than 140/90 mmHg with proteinurea), and abnormal fetal heart rates (either tachycardia or bradycardia).	
Gasiorowska (2020) [70]	Yes	NR	No	No	NR	NR	NR	NR	NR	chromosomal or structural abnormalities and treatment with aspirin, heparin or antihypertensive drugs before enrolment	NR
McLaren (2020) [71]	NR	NR	NR	NR	NR	Yes	Yes	NR	NR	fetal anomalies, known chromosomal defects, known fetal anemia, Rh isoimmunizations, known fetal growth restriction, presence of Parvovirus infection, maternal drug use	NR

Bachani (2020) [72]	Yes	NR	Yes	NR	NR	NR	NR	NR	NR	pre-existing medical disorders including Pre-pregnancy Diabetes, pregnancies complicated by Antepartum Haemorrhage/Haemolytic disorders, pregnancies with foetal congenital abnormalities or chromosomal disease, difference of >6 days in period of gestation estimated by crown rump length on ultrasound to that of calculated from last menstrual period, pregnancy with auto immune conditions, maternal addictions like use of alcohol/tobacco during pregnancy, pregnancy with intrauterine death, neonates requiring NICU admission.	NR
Tenenbaum-Gavish (2020) [73]	Yes	No	No	NR	Yes	NR	No	NR	NR	detection of fetal aneuploidies or major fetal anomalies, increased nuchal translucency thickness >3.5 mm or treatment with aspirin prior to enrollment, ending of pregnancy in termination, miscarriage, or fetal death before 24 weeks' gestation, those who delivered neonates with birthweight below the 5th percentile for gestational age [small for gestational age - SGA]	NR

										and those who delivered before 37 weeks' gestation for reasons other than GDM, pre-eclampsia, or SGA.	
Lehtoranta (2020) [74]	Yes	No	No	No	No	NR	No	No	No	NR	age <35 years, singleton pregnancy and a normal fetal anatomic survey at around 20 weeks of gestation
Phadungkiat wattana (2021) [75]	Yes	No	NR	NR	NR	Yes	Yes	Yes	No	maternal age < 18-years, connective tissue disease, fetal congenital anomalies, and fetal growth restriction	NR
Wei (2021) [18]	Yes	No	NR	Yes	NR	Yes	NR	Yes	NR	Combined with brain, liver and other organ tissue diseases, mental or other cognitive impairment or refused to cooperate with the experiment, uterine organic disease	Age ≥ 20 years old, singleton pregnancy prooved by B-ultrasound
Zhang (2021) [76]	NR	Yes	Yes	NR	NR	Yes	Yes	Yes	NR	mental illness, consuming alcohol, withdrawal from this experiment due to personal reasons	The primipara who did not take drugs that interfered with glucose and fat metabolism younger than 45 years old with clear consciousness
Alanyali (2021) [77]	NR	NR	NR	NR	NR	NR	NR	NR	NR	NR	NR
Liu (2021) [79]	No	NR	No	No	NR	NR	NR	NR	No	NR	NR
Fatihoglu (2021) [80]	Yes	NR	NR	NR	Yes	NR	NR	NR	NR	DM Type 1 or 2, fetal abnormality detected at US, abnormal double and/or triple test result, coexisting morbidities (thyroidal disorders, coagulation problems, etc.)	NR

Chen (2021) [81]	Yes	No	Yes	NR	NR	Yes	Yes	NR	NR	Pre-gestational diabetes, structural or chromosomal fetal anomalies, evidence of fetal infection, and maternal chronic disease other than diabetes mellitus. Fetuses with poor ultrasound image quality or without available fetal cord blood were excluded.	NR
Ali Hssan (2021) [82]	Yes	NR	NR	NR	No	NR	NR	NR	NR	Gestational age more than 40 weeks, pregnant women within the 1st and 2nd trimester, pregnancy with any structural congenital fetal anomaly.	NR
Jamal (2021) [83]	Yes	NR	NR	Yes	Yes	NR	NR	NR	NR	Pre-gestational diabetes mellitus, use of alcohol/cigarettes, IUGR, or other well-known conditions affecting fetal blood flow.	women aged 18-40 years with a singleton viable pregnancy and gestational age from 37 to 40 wk
Perez-Martin (2022) [84]	Yes	No	Yes	NR	NR	NR	NR	NR	NR	history of pregestational diabetes, thyroid disorders, and fetal anomaly	Inclusion criteria were the completion of glucose screening according to the two-step criteria of the National Diabetes Data Group that have been accepted by the Spanish Group of Diabetes in Pregnancy14 and a signed consent form to participate in the study
Chatzakis (2022) [85]	NR	NR	Yes	NR	NR	NR	Yes	NR	NR	Pre-existing DM type 1 or type 2, chromosomally abnormal fetuses with or without structural defects, fetal growth restriction	Pregnant women between 24 and 28gw

Karaca Kutulmus (2022) [86]	Yes	No	NR	Yes	Yes	NR	Yes	Yes	NR	small for gestational age fetus or fetal growth restriction, coexisting systemic diseases, such as pre-gestational diabetes mellitus, connective tissue disorders, and history of alcohol consumption	NR

Table S5. Characteristics of newborns reported in included studies

Author, year	CASES				CONTROLS			
	Gender (%) M/F	Birth weight (g)[a]	APGAR 1st min	APGAR 5th min	Gender M/F (%)	Birth weight (g)	APGAR 1st min	APGAR 5th min
Olofsson (1987) [29]	NR	NR	NR	NR	NR	NR	NR	NR
Landon (1989) [30]	NR	NR	NR	NR	NR	NR	NR	NR
Friedman (1989) [31]	NR	NR	NR	NR	NR	NR	NR	NR
Brown (1990) [32]	57 % /43%	2938±598	NR	NR	58%/42%	3355±536	NR	NR
Johnstone (1992) [33]	NR	NR	NR	NR	NR	NR	NR	NR
Zimmermann (1992) [34]	NR	Birthweight >25th and ≤90th percentile n=29<br>Birthweight≤25th percentile n=5<br>Birthweight ≥90th n=13	NR	NR	NR	NR	NR	NR
Pachi (1993) [35]	NR	NR	NR	NR	NR	NR	NR	NR
Gagnon (1994) [36]	NR	GDM<br>3.514 (2.340-4.620) mean (range)<br>Overt DM<br>3.373 (2.400 - 4.210), mean (range)	NR	<7 n=0 in GDM<br><7 n=0 in Overt DM	NR	3.674 (2.600-4.410)<br>mean (range)	NR	<7 n=0
Weber (1994) [37]	NR	3728±520	NR	NR	NR	3466±485	NR	NR
Santolaya (1994) [38]	NR	3 400±400	NR	NR	NR	3.3±0.2 kg - 1st control<br>2.9±0.5kg - 2nd control	NR	NR
Gazzolo (1995) [39]	NR	3205 ± 330 GDM Group A<br>3276 ± 739 GDM Group B	8.4±0.5 GDM Group A<br>7.1±1.4 GDM Group B	9.3±0.5 GDM Group A<br>8.8±0.8 GDM Group B	NR	3349 ± 327	8.6 ± 0.5	9.4 ± 0.5

Saldeen (1996) [40]	NR	LGA n=6 AGA n=14 SGA n=1	7.5±0.4 mean±se	9.2±0.2 mean±se	NR	LGA n=3 AGA n=7 SGA n=0	7.4±0.5 mean±se	9.6±0.2 mean±se
Grunewald (1996) [41]	NR	3638 (2520-4915) med (range)	NR	<7 n=2	NR	3630 (2855-4540) med (range)	NR	<7 n=0
Weiner (1996) [42]	NR	3719±573 mean±2sd, Diabetes class A 3668±701 mean±2sd, Diabetes class B-R	NR	<7 n=5 in Diabetes class A and n=1 in Diabetes class B-R	NR	3435±432 mean±2sd	NR	<7 n=2
Ursem (1999) [43]	NR	3750 (1440–4310) med (range)	NR	NR	NR	3400 (2720–4330) med (range)	NR	NR
Boito (2003) [44]	NR	3377 (1250– 4830) mean (range)	NR	NR	NR	3280 (2410 - 4220) mean (range)	NR	NR
Tan (2005) [45]	54%/56%	3110 ±70	NR	<7 n=0	NR	3.060±70	NR	<7 n=0
Florio (2006) [46]	NR	4311±56 mean±se	<7 n=1	<7 n=0	NR	3344±66 mean±se	<7 n=0	<7 n=0
Girsen (2008) [47]	NR	3,682±555 Group 1 4,032±559 Group 2	NR	9 (8-10)	NR	3,548 ± 418, gr, (mean ±SD)	NR	9 (7-10)
Russell (2009) [48]	NR	3,805±416	9	9	NR	3,568±486	9	9
To (2009) [49]	NR	3249±465	<4 n=0	<7 n=0	NR	3137±421	<4 n=0	<7 n=0
Parlakgumus (2010) [50]	NR	3141±617	NR	NR	NR	3307±74	NR	NR
Turan (2011) [51]	NR	NR	NR	NR	NR	NR	NR	NR
Nanda (2012) [52]	NR	67.9 (38.3-89.2) percentiles med(IQR)	NR	NR	NR	50.6 (30.9-67.0) percentiles med (IQR)	NR	NR
Fouda (2013) [53]	NR	3136±218	NR	<7 n=3	NR	2861±152	NR	<7 n=0

Suranyi (2013) [54]	NR	Estimated fetal weight DM 1737±1222 GDM 1723±1007	NR	NR	NR	Estimated fetal weight 1223 ± 195.1, gr, (mean ± SD)	NR	NR
Savvidou (2013) [55]	NR	58.3 ± 31.0 percentiles med (IQR)	NR	NR	NR	47.8 ± 29.2 percentiles med (IQR)	NR	NR
Li (2014) [56]	55.3 %/44.7%	3301.06±408.30	8.88±0.46	9.94±0.29	52.6%/47.4%	3266.52±430.25	8.92±0.54	9.95±0.27
Gonzales Gonzales (2014) [57]	NR	3610.1±694	NR	9.0 (9.0-9.0) med (min-max)	NR	3138.5±545	NR	9.0 (9.0-10.0) med (min-max)
Shabani Zanjani (2013) [21]	NR	NR	NR	NR	NR	NR	NR	NR
Moran (2014) [58]	35.9%/64.1%	3481 (2630 – 4900) med (range)	< 7 n=0	7 n=0	32.8%/ 67.2%	3624 (2490 – 5330) med (range)	<7 n=8	< 7 n=0
Bhorat (2014) [59]	NR	NR	NR	NR	NR	NR	NR	NR
Pala (2015) [60]	NR	NR	NR	NR	NR	NR	NR	NR
Liu (2016) [61]	54.43%/44.47%	4010.05±455.16	NR	NR	54.35 %/45.43 %	3345.42±377.54	NR	NR
Peixoto (2016) [14]	NR	3152±546	8.5±1.2	9.2±1.0	NR	2860±681	8.5±1.2	9.3±0.9
Farshchian (2017) [62]	NR	NR	NR	NR	NR	NR	NR	NR
Bugatto (2017) [63]	NR	3169±459	NR	NR	NR	3250±301	NR	NR
Sweeting (2017) [64]	48.8%/51.2% Total 59.6%/40.4% Early GDM 45.7%/54.3% Standard GDM	3307±448 Total 3294±464 Early GDM 3303±440 Standard GDM	NR	NR	48.2%/51.8%	3492±462	NR	NR
Meiramova (2018) [65]	NR	3710±874.6	NR	NR	NR	3676±540	NR	NR
Moodley (2018) [66]	NR	Estimated fetal weight at assessment of indices 529.5±271.8	NR	NR	NR	Estimated fetal weight at assessment of	NR	NR

						indices 517.8±221.9		
Wong (2018) [67]	161.3%/38.7%	3020.32±457.36	9.16±0.90	9.58±0.62	48.4%/51.6%	3107.29±382.07	9.31±0.86	9.72±0.50
Dantas (2019) [68]	NR	NR	NR	NR	NR	NR	NR	NR
Bhorat (2019) [69]	NR	NR	NR	NR	NR	NR	NR	NR
Gasiorowska (2020) [70]	NR	3388±596	NR	NR	NR	3314±512	NR	NR
McLaren (2020) [71]	NR	NR	NR	NR	52%/48%	1308±992	NR	Nr
Bachani (2020) [72]	NR	2993.84±443.79	NR	NR	NR	2653.12± 21.14	NR	NR
Tenenbaum-Gavish (2020) [73]	42.1%/57.9%	3242 (3032–3452) mean (95%CI)	NR	<7 n=0	56.8%/43.2%	3260 (3199–3321) mean (95%CI)	NR	<7 n=0
Lehtoranta (2020) [74]	NR	3686±599	NR	NR	NR	3505±556	NR	NR
Phadungkiatwattana (2021) [75]	57.2%/42.8%	3187.3±427.7	NR	NR	55.7%/44.3%	3161.5±354.1	NR	NR
Wei (2021) [18]	NR	NR	NR	NR	NR	NR	NR	NR
Zhang (2021) [76]	NR	4114±547	NR	NR	NR	3281±429	NR	NR
Alanyali (2021) [77]	NR	NR	NR	NR	NR	NR	NR	NR
Mecacci (2021) [78]	NR	NR	NR	NR	NR	NR	NR	NR
Liu (2021) [79]	54.6%/45.4%	4010.05±455.16	< 7 n=3	<7 n=13	52.2%/47.8%	3345.42±377.54	< 7 n=23	<7 n=1
Fatihoglu (2021) [80]	55%/45%	NR	NR	NR	45.9%/54.1%	NR	NR	NR
Chen (2021) [81]	60.0%/40.0%	3.220±420	NR	NR	151.6%/48.4%	3.280±320	NR	NR
Ali Hssan (2021) [82]	NR	2775±133	NR	NR	NR	2378.8±103.0	NR	NR
Jamal (2021) [83]	NR	NR	NR	NR	NR	NR	NR	NR

Perez-Martin (2022) [84]	42.2%/57.8%	3231±622	NR	NR	54.2%/45.8%	NR	NR	NR
Chatzakis (2022) [85]	NR	NR	NR	NR	NR	NR	NR	NR
Karaca Kutulmus (2022) [86]	NR	NR	NR	NR	NR	NR	NR	NR

Study or Subgroup	DM Mean	DM SD	DM Total	non-DM Mean	non-DM SD	non-DM Total	Weight	Std. Mean Difference IV, Random, 95% CI	Year
Olofsson, 1987	0.94	0.21	40	0.82	0.14	21	3.8%	0.63 [0.09, 1.17]	1987
Pachi, 1993	1.24	0.19	10	0.92	0.12	59	2.7%	2.40 [1.62, 3.19]	1993
Grunewald, 1996	1.04	0.54	24	0.92	0.2	25	3.7%	0.29 [-0.27, 0.86]	1996
Saldeen, 1996	0.84	0.06	21	0.76	0.06	10	2.5%	1.30 [0.47, 2.13]	1996
Ursem, 1999	1.47	0.24	16	1.5	0.39	16	3.1%	-0.09 [-0.78, 0.60]	1999
Boito, 2003	1.1	0.2	32	1.2	0.3	32	4.1%	-0.39 [-0.88, 0.11]	2003
Florio, 2006	0.9	0.47	13	0.61	0.63	40	3.4%	0.48 [-0.15, 1.11]	2006
To, 2009	0.98	0.19	78	1.04	0.17	62	5.1%	-0.33 [-0.66, 0.01]	2009
Shabani Zanjani, 2013	0.91	0.24	33	0.93	0.13	33	4.2%	-0.10 [-0.59, 0.38]	2013
Li, 2014	1.17	0.21	226	1.18	0.21	519	6.0%	-0.05 [-0.20, 0.11]	2014
Pala, 2015	0.93	0.23	39	1.01	0.21	42	4.4%	-0.36 [-0.80, 0.08]	2015
Liu, 2016	0.76	0.12	147	0.7	0.11	124	5.6%	0.52 [0.27, 0.76]	2016
Peixoto, 2016	1	0.1	49	1	0.3	460	5.3%	0.00 [-0.29, 0.29]	2016
Tenenbaum-Gravish, 2020	1.72	0.83	20	1.63	0.76	185	4.3%	0.12 [-0.34, 0.58]	2020
Bachani, 2020	1	0.15	31	1.07	0.33	40	4.3%	-0.26 [-0.73, 0.21]	2020
Lehtoranta, 2020	0.73	0.41	33	0.88	0.2	67	4.5%	-0.52 [-0.94, -0.10]	2020
Alanyali, 2021	1.22	0.47	30	1.32	0.57	30	4.0%	-0.19 [-0.70, 0.32]	2021
Ali Hssan, 2021	1.05	0.23	25	0.9	0.23	25	3.7%	0.64 [0.07, 1.21]	2021
Chen, 2021	0.87	0.14	30	0.82	0.12	31	4.0%	0.38 [-0.13, 0.89]	2021
Fatihoglu, 2021	1.1	0.57	60	1.14	0.62	61	5.0%	-0.07 [-0.42, 0.29]	2021
Jamal, 2021	0.92	0.25	123	0.88	0.19	123	5.6%	0.18 [-0.07, 0.43]	2021
Phadungkiatwattana, 2021	0.89	0.17	138	0.9	0.18	149	5.7%	-0.06 [-0.29, 0.17]	2021
Perez-Martin, 2022	0.95	0.17	56	0.97	0.16	65	4.9%	-0.12 [-0.48, 0.24]	2022
**Total (95% CI)**			**1274**			**2219**	**100.0%**	**0.12 [-0.05, 0.29]**	

Heterogeneity: Tau² = 0.12; Chi² = 96.01, df = 22 (P < 0.00001); I² = 77%
Test for overall effect: Z = 1.37 (P = 0.17)

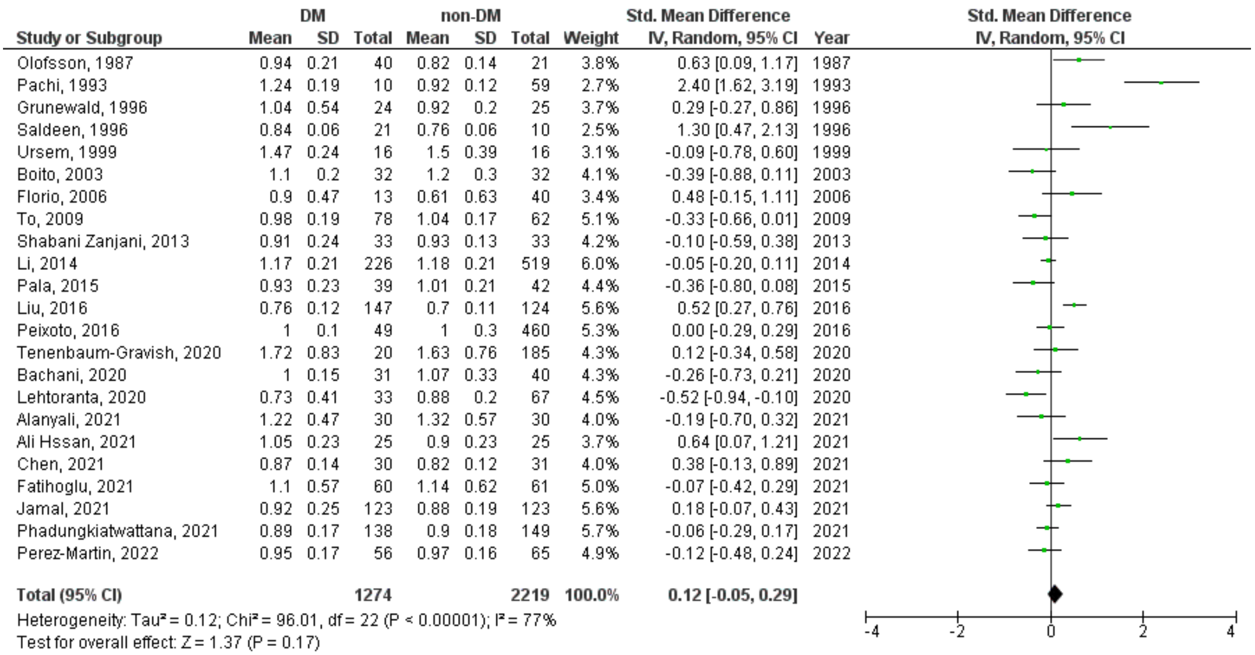

Figure S17. UA-PI Doppler index in pregnant women with vs. pregnant women without diabetes mellitus.

Study or Subgroup	DM Mean	DM SD	DM Total	non-DM Mean	non-DM SD	non-DM Total	Weight	Std. Mean Difference IV, Random, 95% CI	Year
Landon, 1989	2.18	0.22	35	2.49	0.28	117	11.1%	-1.15 [-1.55, -0.75]	1989
Brown, 1990	2.25	1.02	44	2.65	0.66	167	11.5%	-0.53 [-0.87, -0.20]	1990
Weber, 1994	2.95	0.77	9	3.02	0.71	11	7.5%	-0.09 [-0.97, 0.79]	1994
Shabani Zanjani, 2013	2.63	0.28	33	2.54	0.31	33	10.5%	0.30 [-0.18, 0.79]	2013
Li, 2014	3.52	0.75	226	3.5	0.77	519	12.4%	0.03 [-0.13, 0.18]	2014
Liu, 2016	2.16	0.29	147	2.23	0.26	124	12.1%	-0.25 [-0.49, -0.01]	2016
Wei, 2021	3.66	1.08	76	2.49	0.63	76	11.4%	1.32 [0.97, 1.67]	2021
Fatihoglu, 2021	3.8	0.75	60	3.7	0.15	61	11.4%	0.18 [-0.17, 0.54]	2021
Jamal, 2021	2.49	0.63	123	2.35	0.45	123	12.0%	0.25 [0.00, 0.51]	2021
**Total (95% CI)**			**753**			**1231**	**100.0%**	**0.01 [-0.37, 0.39]**	

Heterogeneity: Tau² = 0.29; Chi² = 106.19, df = 8 (P < 0.00001); I² = 92%
Test for overall effect: Z = 0.05 (P = 0.96)

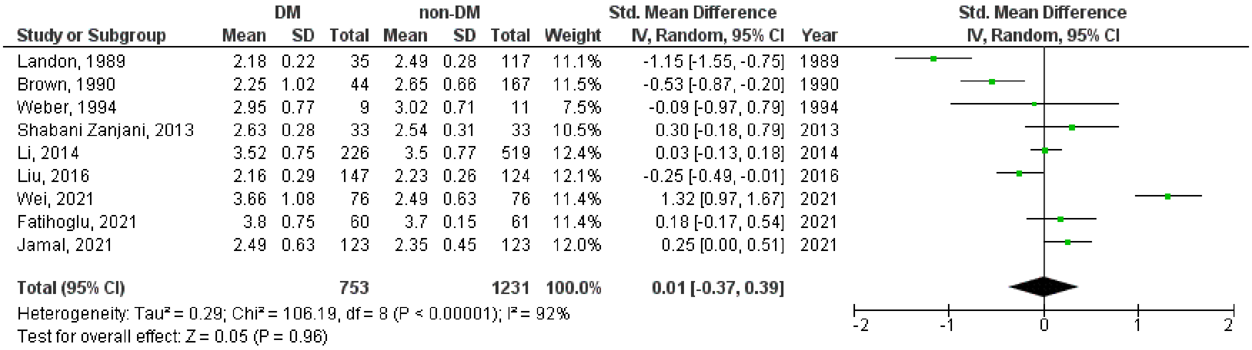

Figure S18. UA-S/D ratio Doppler index in pregnant women with vs. pregnant women without diabetes mellitus.

	DM			non-DM				Std. Mean Difference	
Study or Subgroup	Mean	SD	Total	Mean	SD	Total	Weight	IV, Random, 95% CI	Year
Florio, 2006	0.48	0.61	13	0.46	0.76	40	5.2%	0.03 [-0.60, 0.65]	2006
Sawidou, 2013	1	0.28	1037	0.99	0.29	56649	7.1%	0.03 [-0.03, 0.10]	2013
Shabani Zanjani, 2013	2.07	0.4	33	1.85	0.42	33	5.8%	0.53 [0.04, 1.02]	2013
Liu, 2016	1.29	0.22	147	1.58	0.11	124	6.7%	-1.62 [-1.90, -1.35]	2016
Peixoto, 2016	1.9	0.3	44	1.8	0.4	406	6.5%	0.25 [-0.06, 0.57]	2016
Bachani, 2020	3.82	0.78	31	2.74	0.98	40	5.7%	1.19 [0.68, 1.70]	2020
McLaren, 2020	1.87	0.45	20	1.68	0.4	34	5.5%	0.45 [-0.11, 1.01]	2020
Lehtoranta, 2020	1.67	0.71	33	1.58	0.47	67	6.1%	0.16 [-0.26, 0.58]	2020
Ali Hssan, 2021	0.57	0.16	25	0.68	0.2	25	5.4%	-0.60 [-1.17, -0.03]	2021
Jamal, 2021	1.72	0.42	123	1.73	0.45	123	6.7%	-0.02 [-0.27, 0.23]	2021
Fatihoglu, 2021	1.4	0.27	60	1.4	0.45	61	6.4%	0.00 [-0.36, 0.36]	2021
Liu, 2021	1.09	0.36	1268	1.25	0.34	10922	7.1%	-0.47 [-0.53, -0.41]	2021
Phadungkiatwattana, 2021	1.5	0.37	138	1.49	0.29	149	6.8%	0.03 [-0.20, 0.26]	2021
Zhang, 2021	0.95	0.15	80	0.57	0.24	80	6.3%	1.89 [1.52, 2.26]	2021
Wei, 2021	0.67	0.21	76	0.52	0.16	76	6.5%	0.80 [0.47, 1.13]	2021
Perez-Martin, 2022	1.99	0.4	56	2	0.3	65	6.3%	-0.03 [-0.39, 0.33]	2022
**Total (95% CI)**			**3184**			**68894**	**100.0%**	**0.15 [-0.12, 0.42]**	

Heterogeneity: Tau² = 0.26; Chi² = 455.50, df = 15 (P < 0.00001); I² = 97%
Test for overall effect: Z = 1.08 (P = 0.28)

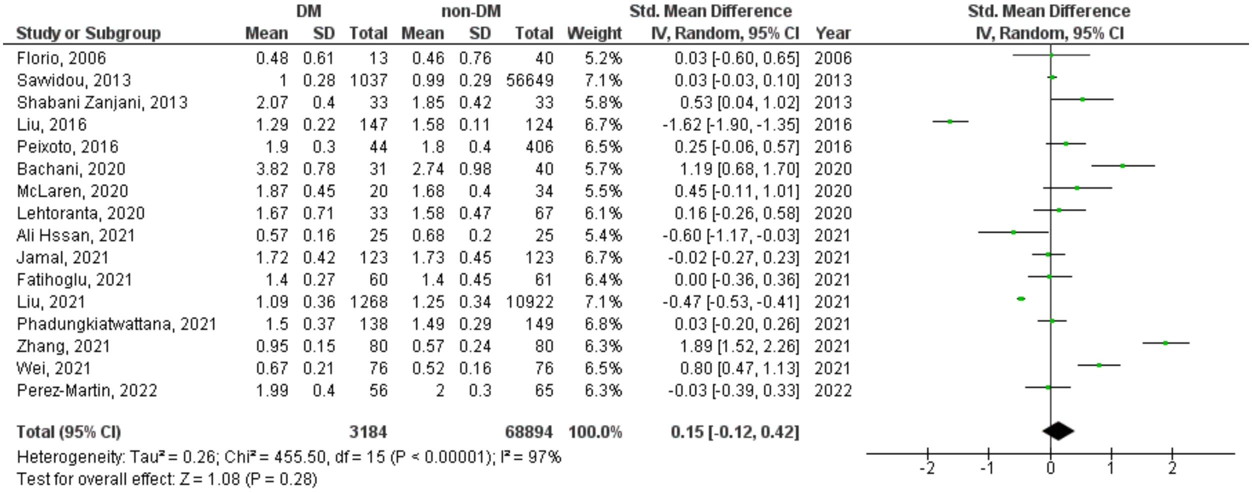

Figure S19. MCA-PI Doppler index in pregnant women with vs. pregnant women without diabetes mellitus.

	DM			non-DM				Std. Mean Difference	
Study or Subgroup	Mean	SD	Total	Mean	SD	Total	Weight	IV, Random, 95% CI	Year
Shabani Zanjani, 2013	0.85	0.05	33	0.84	0.05	33	11.0%	0.20 [-0.29, 0.68]	2013
Fouda, 2013	0.79	0.05	23	0.79	0.05	27	10.9%	0.00 [-0.56, 0.56]	2013
Liu, 2016	0.7	0.06	147	0.78	0.02	124	11.3%	-1.73 [-2.01, -1.45]	2016
Moodley, 2018	0.83	0.09	22	0.84	0.09	23	10.8%	-0.11 [-0.69, 0.48]	2018
Ali Hssan, 2021	0.77	0.06	25	0.75	0.07	25	10.9%	0.30 [-0.26, 0.86]	2021
Fatihoglu, 2021	0.8	0.15	60	0.8	0.15	61	11.2%	0.00 [-0.36, 0.36]	2021
Zhang, 2021	1.94	0.31	80	1.31	0.27	80	11.2%	2.16 [1.77, 2.55]	2021
Wei, 2021	1.41	0.44	76	1.02	0.29	76	11.3%	1.04 [0.70, 1.38]	2021
Jamal, 2021	0.77	0.1	123	0.77	0.08	123	11.4%	0.00 [-0.25, 0.25]	2021
Liu, 2021	0.64	0.17	1268	0.76	0	10922		Not estimable	2021
**Total (95% CI)**			**1857**			**11494**	**100.0%**	**0.21 [-0.57, 0.98]**	

Heterogeneity: Tau² = 1.37; Chi² = 299.95, df = 8 (P < 0.00001); I² = 97%
Test for overall effect: Z = 0.52 (P = 0.60)

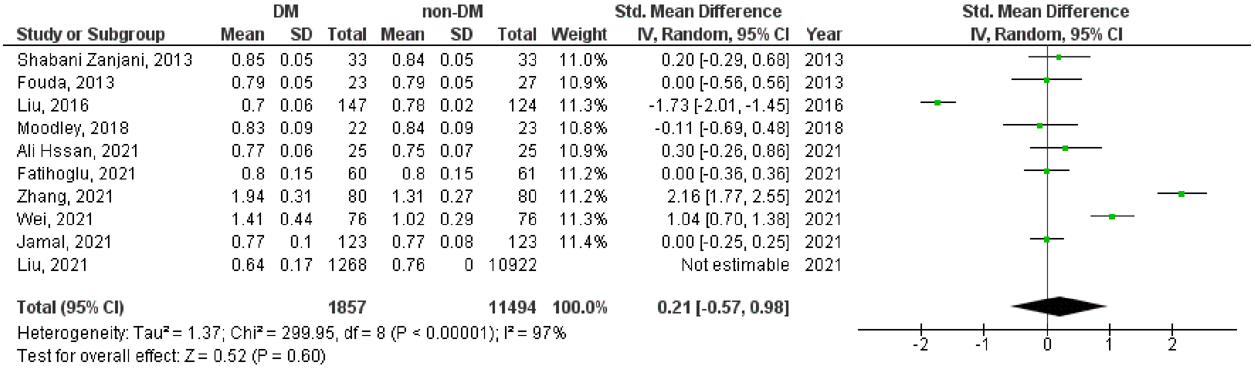

Figure S20. MCA-RI Doppler index in pregnant women with vs. pregnant women without diabetes mellitus.

	DM			non-DM				Std. Mean Difference	
Study or Subgroup	Mean	SD	Total	Mean	SD	Total	Weight	IV, Random, 95% CI	Year
Shabani Zanjani, 2013	7.18	3.55	33	7	2.64	33	16.1%	0.06 [-0.43, 0.54]	2013
Liu, 2016	3.44	0.56	147	4.68	0.45	124	16.7%	-2.41 [-2.73, -2.10]	2016
Jamal, 2021	4.77	1.81	123	4.86	2.02	123	16.9%	-0.05 [-0.30, 0.20]	2021
Fatihoglu, 2021	5.2	0.15	60	5.2	0.25	61	16.6%	0.00 [-0.36, 0.36]	2021
Liu, 2021	3.22	1.03	1268	3.95	0.96	10922	17.1%	-0.75 [-0.81, -0.70]	2021
Wei, 2021	3.55	1.14	76	2.21	0.54	76	16.6%	1.49 [1.13, 1.86]	2021
**Total (95% CI)**			**1707**			**11339**	**100.0%**	**-0.28 [-1.07, 0.51]**	

Heterogeneity: Tau² = 0.94; Chi² = 309.73, df = 5 (P < 0.00001); I² = 98%
Test for overall effect: Z = 0.70 (P = 0.48)

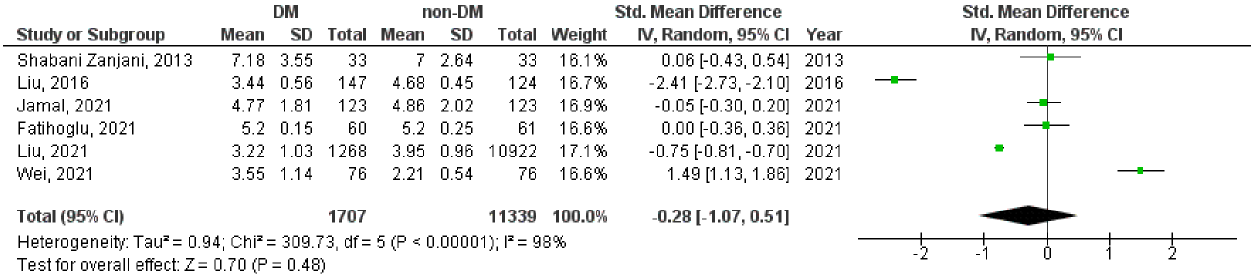

Figure S21. MCA-S/D ratio Doppler index in pregnant women with vs. pregnant women without diabetes mellitus.

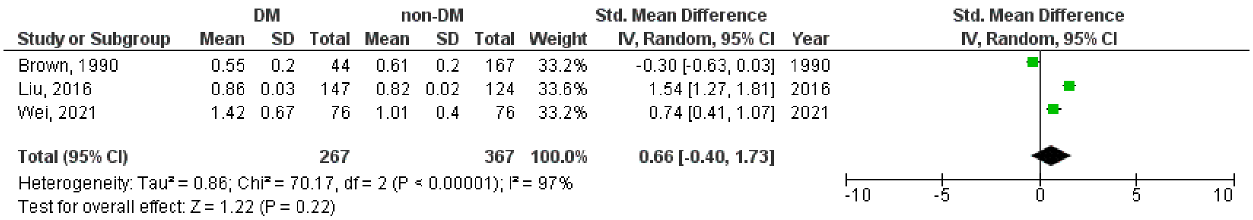

Figure S22. UtA-RI Doppler index in pregnant women with vs. pregnant women without diabetes mellitus.

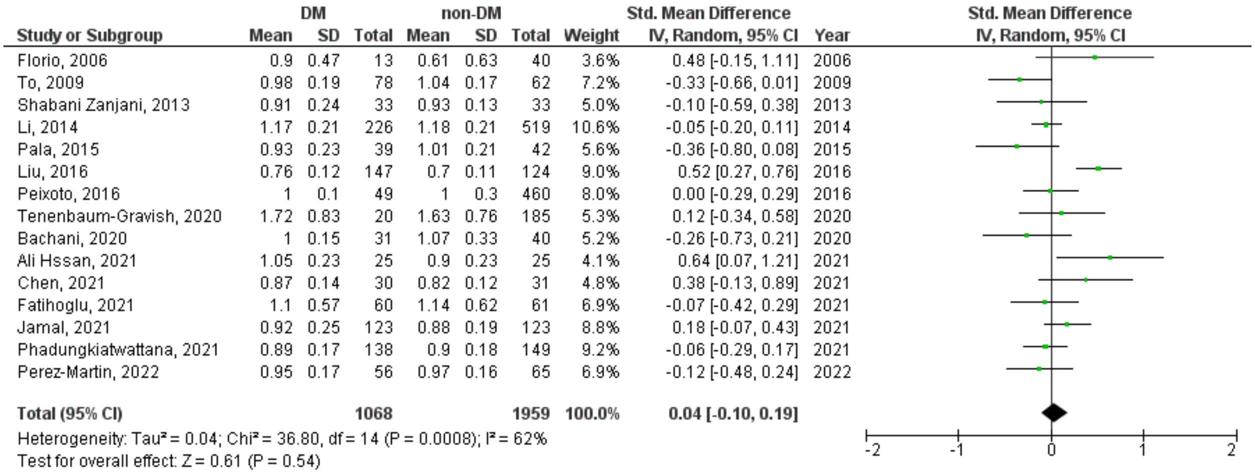

Figure S23. UA-PI Doppler index in pregnant women with GDM vs. women without GDM.

Study or Subgroup	DM Mean	DM SD	DM Total	non-DM Mean	non-DM SD	non-DM Total	Weight	Std. Mean Difference IV, Random, 95% CI	Year
Brown, 1990	0.6	0.12	44	0.63	0.13	167	9.6%	-0.23 [-0.57, 0.10]	1990
Gagnon, 1994	0.61	0.02	20	0.57	0.05	14	5.7%	1.10 [0.37, 1.84]	1994
Gazzolo, 1995	0.6	0.1	61	0.59	0.04	100	9.8%	0.14 [-0.17, 0.46]	1995
Shabani Zanjani, 2013	0.61	0.05	33	0.6	0.05	33	8.0%	0.20 [-0.29, 0.68]	2013
Li, 2014	0.72	0.12	226	0.71	0.09	519	11.2%	0.10 [-0.06, 0.26]	2014
Pala, 2015	1.77	0.5	39	1.94	0.81	42	8.5%	-0.25 [-0.69, 0.19]	2015
Liu, 2016	0.53	0.06	147	0.55	0.05	124	10.5%	-0.36 [-0.60, -0.12]	2016
Ali Hssan, 2021	1.66	0.35	25	1.54	0.38	25	7.3%	0.32 [-0.23, 0.88]	2021
Jamal, 2021	0.58	0.08	123	0.56	0.07	123	10.4%	0.27 [0.01, 0.52]	2021
Wei, 2021	1.53	0.41	76	1.14	0.31	76	9.6%	1.07 [0.73, 1.41]	2021
Fatihoglu, 2021	0.7	0.15	60	0.72	0.17	61	9.4%	-0.12 [-0.48, 0.23]	2021
**Total (95% CI)**			**854**			**1284**	**100.0%**	**0.16 [-0.08, 0.41]**	

Heterogeneity: $Tau^2 = 0.13$; $Chi^2 = 62.36$, df = 10 ($P < 0.00001$); $I^2 = 84\%$
Test for overall effect: Z = 1.32 (P = 0.19)

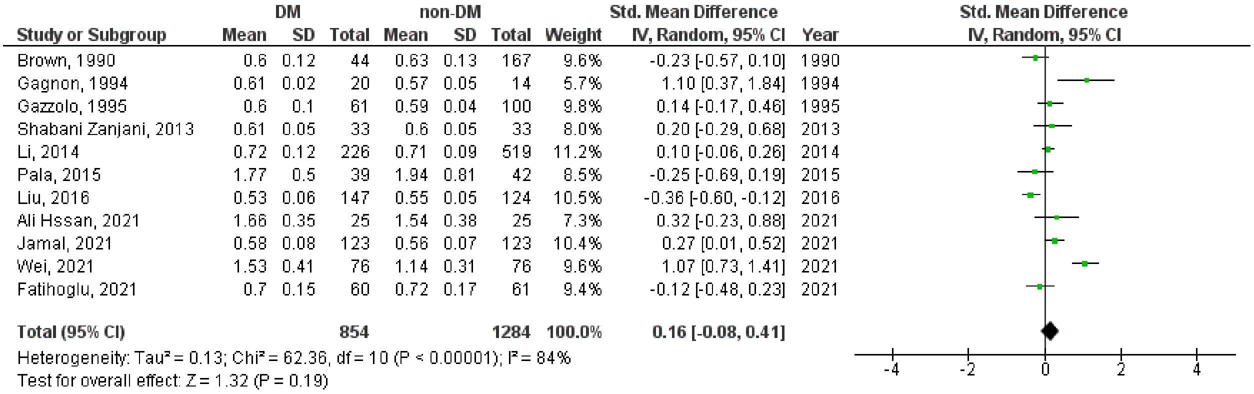

Figure S24. UA-RI Doppler index in pregnant women with GDM vs. women without GDM.

Study or Subgroup	DM Mean	DM SD	DM Total	non-DM Mean	non-DM SD	non-DM Total	Weight	Std. Mean Difference IV, Random, 95% CI	Year
Brown, 1990	2.25	1.02	44	2.65	0.66	167	14.1%	-0.53 [-0.87, -0.20]	1990
Shabani Zanjani, 2013	2.63	0.28	33	2.54	0.31	33	12.5%	0.30 [-0.18, 0.79]	2013
Li, 2014	3.52	0.75	226	3.5	0.77	519	15.6%	0.03 [-0.13, 0.18]	2014
Liu, 2016	2.16	0.29	147	2.23	0.26	124	15.0%	-0.25 [-0.49, -0.01]	2016
Fatihoglu, 2021	3.8	0.75	60	3.7	0.15	61	13.9%	0.18 [-0.17, 0.54]	2021
Wei, 2021	3.66	1.08	76	2.49	0.63	76	14.0%	1.32 [0.97, 1.67]	2021
Jamal, 2021	2.49	0.63	123	2.35	0.45	123	14.9%	0.25 [0.00, 0.51]	2021
**Total (95% CI)**			**709**			**1103**	**100.0%**	**0.18 [-0.19, 0.54]**	

Heterogeneity: $Tau^2 = 0.21$; $Chi^2 = 71.08$, df = 6 ($P < 0.00001$); $I^2 = 92\%$
Test for overall effect: Z = 0.95 (P = 0.34)

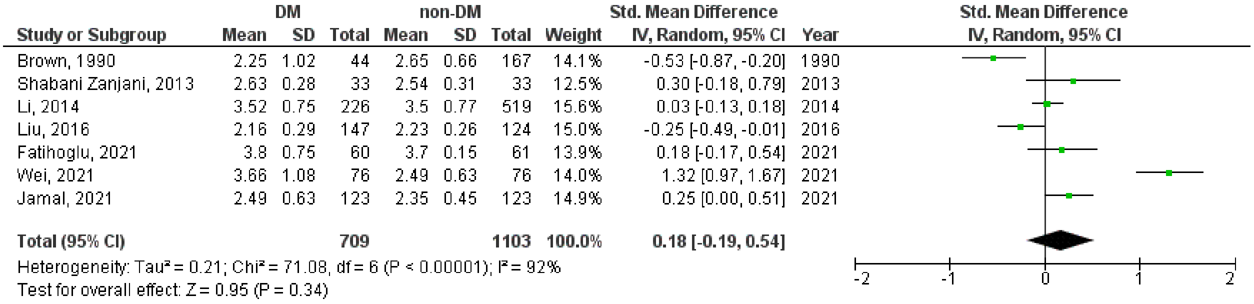

Figure S25. UA-S/D ratio Doppler index in pregnant women with GDM vs. women without GDM.

Study or Subgroup	DM Mean	DM SD	DM Total	non-DM Mean	non-DM SD	non-DM Total	Weight	Std. Mean Difference IV, Random, 95% CI	Year
Florio, 2006	0.48	0.61	13	0.46	0.76	40	5.5%	0.03 [-0.60, 0.65]	2006
Shabani Zanjani, 2013	2.07	0.4	33	1.85	0.42	33	6.2%	0.53 [0.04, 1.02]	2013
Sawidou, 2013	1	0.28	1037	0.99	0.29	56649	7.6%	0.03 [-0.03, 0.10]	2013
Liu, 2016	1.29	0.22	147	1.58	0.11	124	7.1%	-1.62 [-1.90, -1.35]	2016
Peixoto, 2016	1.9	0.3	44	1.8	0.4	406	6.9%	0.25 [-0.06, 0.57]	2016
McLaren, 2020	1.87	0.45	20	1.68	0.4	34	5.8%	0.45 [-0.11, 1.01]	2020
Bachani, 2020	3.82	0.78	31	2.74	0.98	40	6.1%	1.19 [0.68, 1.70]	2020
Jamal, 2021	1.72	0.42	123	1.73	0.45	123	7.2%	-0.02 [-0.27, 0.23]	2021
Ali Hssan, 2021	0.57	0.16	25	0.68	0.2	25	5.8%	-0.60 [-1.17, -0.03]	2021
Phadungkiatwattana, 2021	1.5	0.37	138	1.49	0.29	149	7.2%	0.03 [-0.20, 0.26]	2021
Fatihoglu, 2021	1.4	0.27	60	1.4	0.45	61	6.8%	0.00 [-0.36, 0.36]	2021
Liu, 2021	1.09	0.36	1268	1.25	0.34	10922	7.6%	-0.47 [-0.53, -0.41]	2021
Wei, 2021	0.67	0.21	76	0.52	0.16	76	6.9%	0.80 [0.47, 1.13]	2021
Zhang, 2021	0.95	0.15	80	0.57	0.24	80	6.7%	1.89 [1.52, 2.26]	2021
Perez-Martin, 2022	1.99	0.4	56	2	0.3	65	6.8%	-0.03 [-0.39, 0.33]	2022
**Total (95% CI)**			**3151**			**68827**	**100.0%**	**0.15 [-0.13, 0.43]**	

Heterogeneity: Tau² = 0.27; Chi² = 452.99, df = 14 (P < 0.00001); I² = 97%
Test for overall effect: Z = 1.04 (P = 0.30)

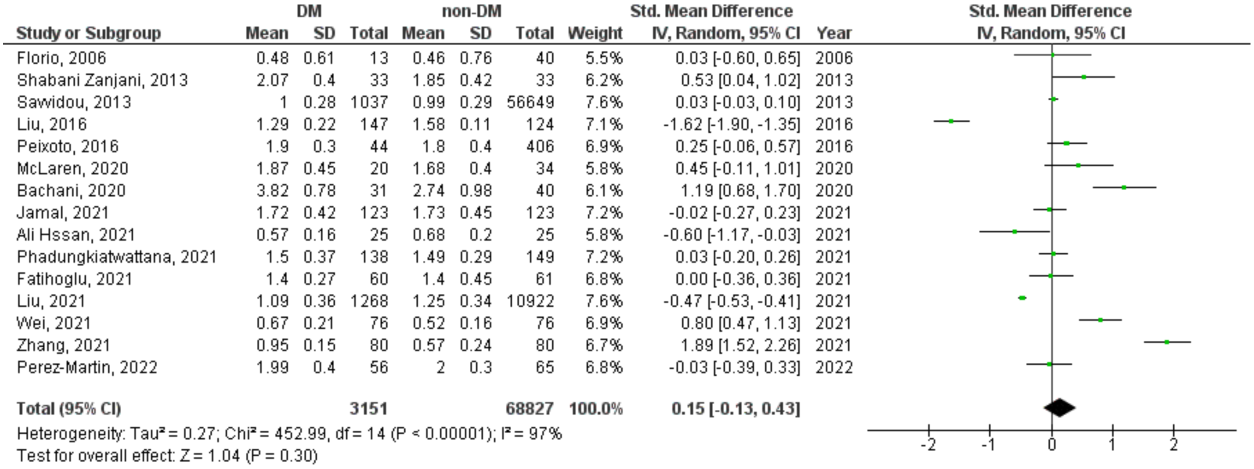

Figure S26. MCA-PI Doppler index in pregnant women with GDM vs. women without GDM.

Study or Subgroup	DM Mean	DM SD	DM Total	non-DM Mean	non-DM SD	non-DM Total	Weight	Std. Mean Difference IV, Random, 95% CI	Year
Shabani Zanjani, 2013	0.85	0.05	33	0.84	0.05	33	14.2%	0.20 [-0.29, 0.68]	2013
Fouda, 2013	0.79	0.05	23	0.79	0.05	27	14.0%	0.00 [-0.56, 0.56]	2013
Liu, 2016	0.7	0.06	147	0.78	0.02	124	14.5%	-1.73 [-2.01, -1.45]	2016
Ali Hssan, 2021	0.77	0.06	25	0.75	0.07	25	14.0%	0.30 [-0.26, 0.86]	2021
Jamal, 2021	0.77	0.1	123	0.77	0.08	123	14.5%	0.00 [-0.25, 0.25]	2021
Wei, 2021	1.41	0.44	76	1.02	0.29	76	14.4%	1.04 [0.70, 1.38]	2021
Zhang, 2021	1.94	0.31	80	1.31	0.27	80	14.3%	2.16 [1.77, 2.55]	2021
Liu, 2021	0.64	0.17	1268	0.76	0	10922		Not estimable	2021
**Total (95% CI)**			**1775**			**11410**	**100.0%**	**0.28 [-0.71, 1.27]**	

Heterogeneity: Tau² = 1.73; Chi² = 299.61, df = 6 (P < 0.00001); I² = 98%
Test for overall effect: Z = 0.55 (P = 0.58)

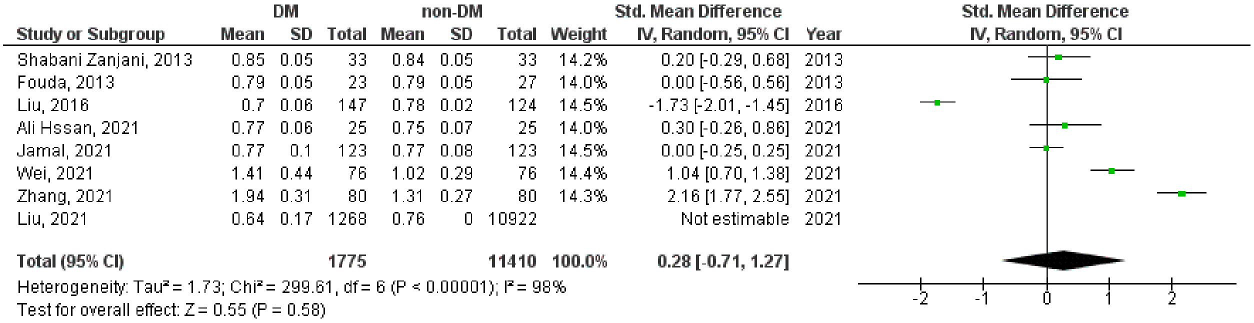

Figure S27. MCA-RI Doppler index in pregnant women with GDM vs. women without GDM.

Study or Subgroup	DM Mean	DM SD	DM Total	non-DM Mean	non-DM SD	non-DM Total	Weight	Std. Mean Difference IV, Random, 95% CI	Year
Shabani Zanjani, 2013	7.18	3.55	33	7	2.64	33	16.1%	0.06 [-0.43, 0.54]	2013
Liu, 2016	3.44	0.56	147	4.68	0.45	124	16.7%	-2.41 [-2.73, -2.10]	2016
Jamal, 2021	4.77	1.81	123	4.86	2.02	123	16.9%	-0.05 [-0.30, 0.20]	2021
Fatihoglu, 2021	5.2	0.15	60	5.2	0.25	61	16.6%	0.00 [-0.36, 0.36]	2021
Liu, 2021	3.22	1.03	1268	3.95	0.96	10922	17.1%	-0.75 [-0.81, -0.70]	2021
Wei, 2021	3.55	1.14	76	2.21	0.54	76	16.6%	1.49 [1.13, 1.86]	2021
**Total (95% CI)**			**1707**			**11339**	**100.0%**	**-0.28 [-1.07, 0.51]**	

Heterogeneity: Tau² = 0.94; Chi² = 309.73, df = 5 (P < 0.00001); I² = 98%
Test for overall effect: Z = 0.70 (P = 0.48)

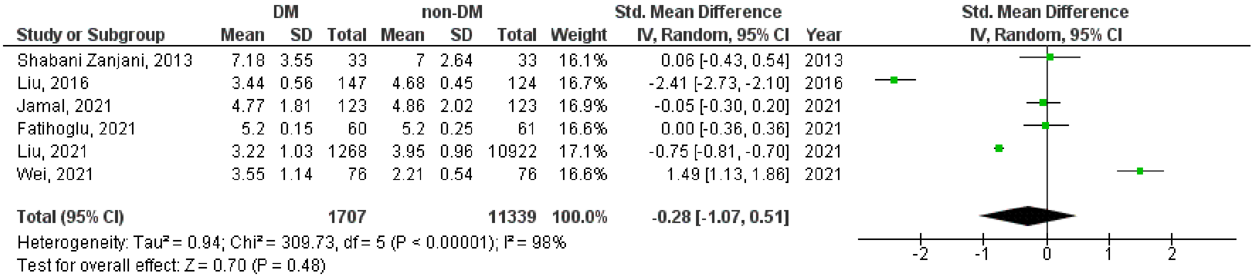

Figure S28. MCA-S/D ratio Doppler index in pregnant women with GDM vs. women without GDM.

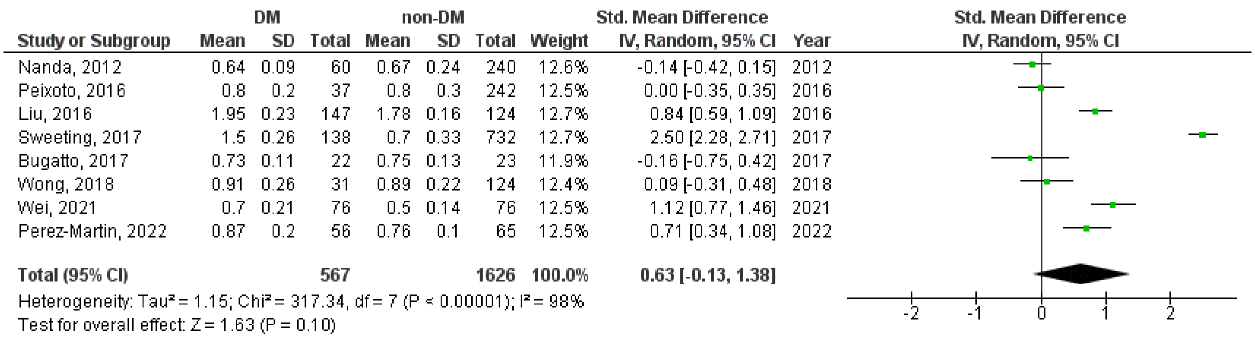

Figure S29. UtA-PI Doppler index in pregnant women with GDM vs. women without GDM.
